# A cookbook for DNase Hi-C

**DOI:** 10.1186/s13072-021-00389-5

**Published:** 2021-03-20

**Authors:** Maria Gridina, Evgeniy Mozheiko, Emil Valeev, Ludmila P. Nazarenko, Maria E. Lopatkina, Zhanna G. Markova, Maria I. Yablonskaya, Viktoria Yu Voinova, Nadezhda V. Shilova, Igor N. Lebedev, Veniamin Fishman

**Affiliations:** 1 Institute of Cytology and Genetics SB RAS, Lavrentjeva ave 10, Novosibirsk, Russia; 2 Novosibirsk State University, Pirogova str., 2, Novosibirsk, Russia; 3 Research Institute of Medical Genetics, Tomsk National Research Medical Center, Kooperativny Str, 5, Tomsk, Russia; 4 Clinical Research Institute of Pediatrics Named After Acad. Y.E. Veltischev, Moscow, Russia; 5 Research Centre for Medical Genetics, Moskvorechie str., 1, Moscow, Russia

**Keywords:** DNAse I, Hi-C, Genome organization, Human peripheral blood, K562, LNCaP, A549

## Abstract

**Background:**

The Hi-C technique is widely employed to study the 3-dimensional chromatin architecture and to assemble genomes. The conventional in situ Hi-C protocol employs restriction enzymes to digest chromatin, which results in nonuniform genomic coverage. Using sequence-agnostic restriction enzymes, such as DNAse I, could help to overcome this limitation.

**Results:**

In this study, we compare different DNAse Hi-C protocols and identify the critical steps that significantly affect the efficiency of the protocol. In particular, we show that the SDS quenching strategy strongly affects subsequent chromatin digestion. The presence of biotinylated oligonucleotide adapters may lead to ligase reaction by-products, which can be avoided by rational design of the adapter sequences. Moreover, the use of nucleotide-exchange enzymes for biotin fill-in enables simultaneous labelling and repair of DNA ends, similar to the conventional Hi-C protocol. These improvements simplify the protocol, making it less expensive and time-consuming.

**Conclusions:**

We propose a new robust protocol for the preparation of DNAse Hi-C libraries from cultured human cells and blood samples supplemented with experimental controls and computational tools for the evaluation of library quality.

**Supplementary Information:**

The online version contains supplementary material available at 10.1186/s13072-021-00389-5.

## Background

The coupling of the chromatin conformation capture technique with next-generation sequencing has resulted in the development of a simple and efficient Hi-C protocol, which enables the genome-wide chromatin architecture to be studied [1, 2]. Along with numerous insights into nuclear organization and dynamics, Hi-C results have demonstrated that spatial contacts between loci strongly depend on the genomic distance between them [1, 2]. In particular, adjacent genomic segments interact considerably more frequently than distal or interchromosomal regions. This dependence of chromatin contacts on genomic distance has been observed in all studied cell types [3–5] and can be utilized to infer the order of scaffolds in poorly assembled genomes, providing chromosome-length assemblies [6–11]. For species with a well-assembled genome, such as humans, the Hi-C technique can be used to detect structural variations, which alter the order of genomic segments and therefore lead to significant changes in chromatin interaction frequencies [6, 12–17]. In addition, one can extract information about single nucleotide variations (SNVs) from Hi-C reads. The studies described in [18, 19] have shown that coupling proximity information and SNV data can be used to phase genomes, and we have recently suggested using a cognate approach for genetic diagnostics [20].

Classical Hi-C protocols rely on restriction enzymes for fragmenting genomic DNA [1, 2]. This fragmentation limits the theoretical resolution of Hi-C analysis by the restriction fragment length and results in nonuniform genomic coverage biased towards the regions flanking the restriction enzyme recognition sites. For most genome-wide analyses of chromatin architecture, this limitation is not essential because achieving a resolution beyond the restriction fragment length would require an extremely high sequencing depth. However, for capture-Hi-C data, as well as for scaffolding or genotyping applications, high resolution and uniform coverage are desirable.

Several solutions have been proposed to overcome these limitations. First, 4-cutter enzymes are currently used to prepare Hi-C libraries, which decreases the average fragment length compared to the 6-cutter enzymes used previously [2]. Moreover, a combination of several restriction enzymes can be utilized to decrease fragment length. However, the distribution of cut sites in these cases is not uniform, and there are always some genomic regions that are not well represented in a Hi-C dataset prepared using restriction enzymes.

Second, nucleases that have no sequence-specific cutting preferences, such as DNase I [21–23] or MNase [24, 25], can be used in the Hi-C protocol. MNase has recently been utilized to prepare high-resolution whole-genome Hi-C datasets of yeasts and humans [24, 25]. At the same time, the Zhijun Duan group has developed a protocol for the preparation of capture- and genome-wide Hi-C datasets using DNase I [21–23]. Providing uniform coverage and a theoretically unlimited resolution of data, these protocols seem to be the most suitable when using Hi-C sequencing for genotyping purposes.

In this study, we aimed to optimize the DNase Hi-C protocol to allow efficient capture of chromatin interactions in human cells. We showed that optimization of the cell lysis and chromatin digestion conditions by DNase I was critical for the preparation of high-quality libraries. We also compared two different strategies for DNA end-labelling: the original strategy employing biotinylated linkers and an alternative strategy relying on nucleotide exchange. Based on our observations, we suggested several controls that enabled us to estimate library quality before and after sequencing. Finally, we showed that Burrows-Wheeler Aligner could efficiently map chimaeric reads produced both in the presence and absence of biotinylated linkers and provided bioinformatic tools and pipelines suitable for the analysis of DNase Hi-C data.

## Results

We started our study by benchmarking the published DNAse Hi-C protocol developed by Ma et al. [22]. For this benchmarking, we reanalysed published data and applied the protocol without modifications to a collection of human blood samples and K562 cells. Through the manuscript, we label the reanalysis of the original data as “Ma et al. (reanalysed)”, whereas new assays following the original protocol are labelled as “Protocol: Ma et al.”. We follow the same naming conventions for another protocol published by Ramani et al. [21], which we also benchmarked. When referring to the modified versions of these protocols developed in this study, we highlight key modifications in the protocol title, for example, “Protocol: Ramani et al. long linker”. We list all the protocols used in this study in Table 1.

**Table 1:** DNAse Hi-C protocols used in this study

Protocol name	Short protocol description
Ma et al. (reanalysed)	Data from Ma et al. [22] reanalysed using our pipeline
Ramani et al. (reanalysed)	Data from Ramani et al. [21] reanalysed using our pipeline
Protocol Ma et al.	Reproduction of the protocol from [22] in our laboratory
Protocol Ma et al. new blunt	Modification of the protocol from [22]. We changed the blunt-adapter sequences to prevent the formation of adapter multimers
Protocol Ma et al. long linker	Modification of the protocol from [22]. We abolished the two-step ligation procedure. We used the long linker derived from the BAT-Hi-C protocol instead
Protocol Ramani et al	Reproduction of the protocol from [21] in our lab
Protocol Ramani et al. long linker	Modification of the protocol from [21]. We abolished the two-step ligation procedure and used the long linker derived from the BAT-Hi-C protocol instead
Protocol Ramani et al. biotin fill-in	Modification of the protocol from [21]. We abolished the two-step ligation procedure. End-labelling with biotinylated dCTP nucleotides was performed during the end-repair step following DNase treatment

**Fig. 1:**
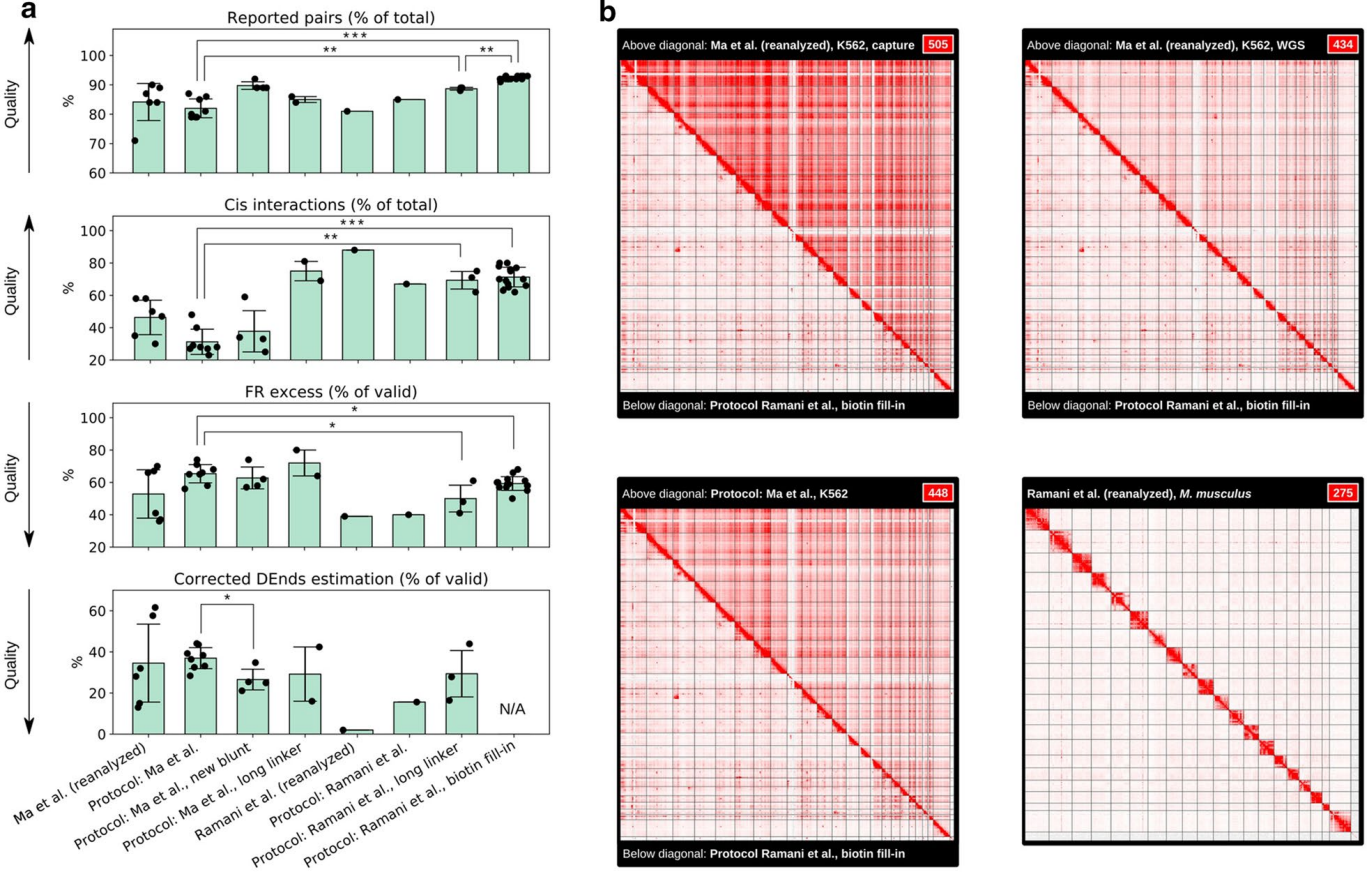
Improving the DNAse Hi-C protocol allows the generation of high-quality Hi-C maps. **a** Quality metrics of Hi-C datasets. Data are grouped according to the protocol employed for library construction. Each dot represents an independent Hi-C library preparation. The dataset names are explained in Table 1, and all the details of each protocol are described in the “Methods” section. Note that Ramani et al. ([21]) performed Hi-C on mouse samples, whereas other data were from human cells, which could explain some of the differences between “Ramani et al. (reanalysed)” and other samples. The reported pairs percentage indicates the mapping efficiency; *cis*-interactions reflect noise levels; FR-excess indicates the overrepresentation of reads in the forward–reverse (inward) orientation, a signature of undigested or unligated DNA (DEnds); when possible, estimated DEnds were corrected using information about biotinylated linker incorporation (see “Methods” section) and are shown on the corrected DEnds estimation plot. The significance of differences between groups was estimated using the Mann–Whitney test. **b** Representative Hi-C data obtained using different protocols for human K562 cells and for mouse brain cells (in the case of reanalysed Ramani et al. data). For K562 cells, each Hi-C heatmap shows a comparison between results obtained using the protocol from [23] (above the diagonal line) and data obtained using the biotin fill-in protocol developed in this study (below the diagonal line). All data were downsampled to the same sequencing depth, and the number in the top right corner indicates the values selected on the Juicebox colour slider. Additional heatmaps showing representative genomic regions are shown in Additional file 1: Fig. AS1

Our first attempts to use the DNase Hi-C protocol by Ma et al. resulted in low-quality libraries (Fig. 1a, b; Additional file 1: Table S1). In particular, we found a large proportion of interchromosomal (*trans*) interactions, indicating high-level random ligations. There was also a large excess of read pairs in the inward (forward–reverse) orientation (also called “dangling ends” or DEnds), suggesting low digestion and/or ligation efficiency. Moreover, there were overrepresented sequences corresponding to ligation products between the oligonucleotide adapters used for DNA end-labelling. Finally, a high percentage of reads failed to align due to the presence of adapter multimers. To optimize the protocol, we prepared and sequenced a few dozen DNase Hi-C libraries. By trial and error, we identified several critical steps that significantly affected the efficiency of the protocol (see Fig. 1a for a comparison of the protocol quality metrics and Fig. 1b and Additional file 1: Fig. S1 for representative Hi-C maps). We summarized our experience as a set of hints and quality controls supplemented with a detailed protocol and representative results.

## Hint 1. Digest the chromatin properly

### Problem

**Fig. 2:**
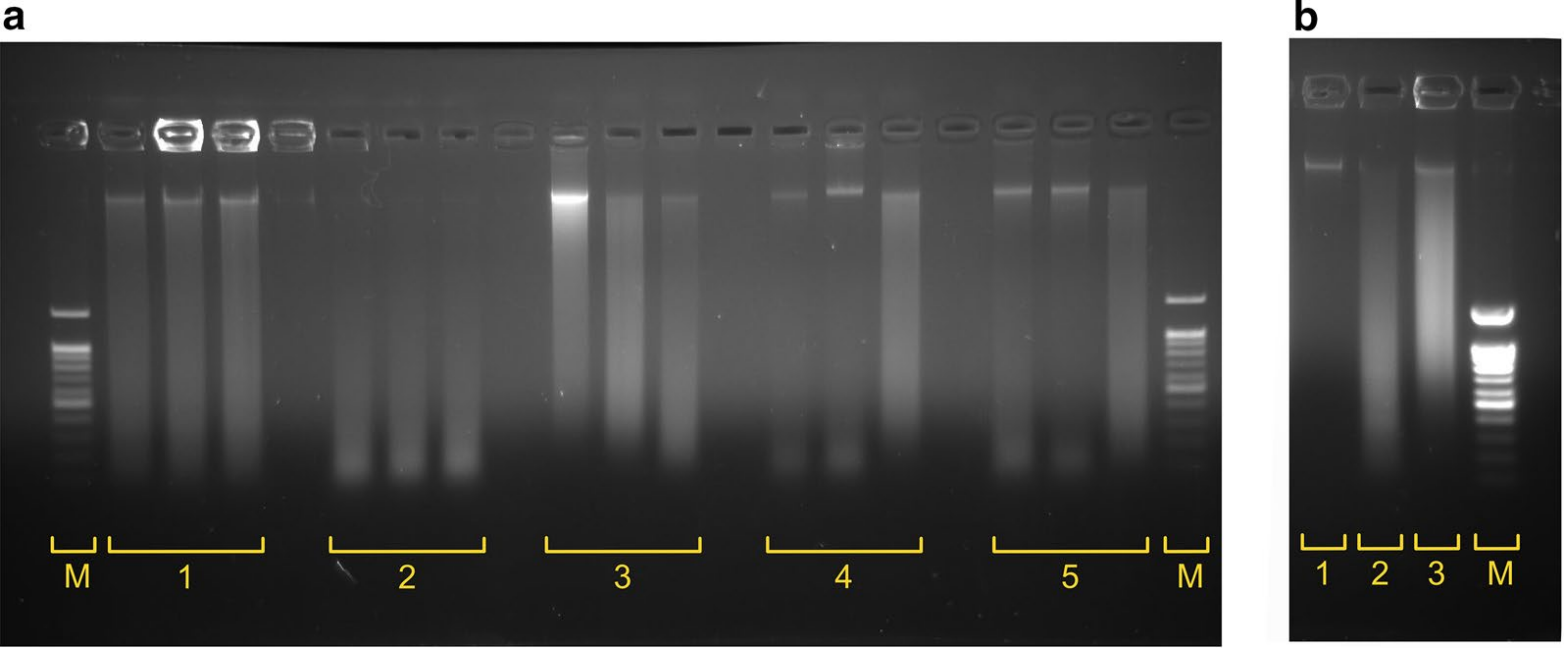
Chromatin digestion and ligation in DNAse Hi-C experiments. **a**) Reproducibility of chromatin digestion by DNase I under the conditions recommended by Ramani et al. [21] and Ma et al. [22] assessed in three independent replicas. 1—Ramani et al., 1 U DNase I. 2—Ramani et al., 2 U DNase I. 3—Ma et al., 2 U DNase I. 4—Ma et al., 4 U DNase I. 5—Ma et al., 6 U DNase I. Lane M shows a 100 bp DNA ladder. **b** Intermediate controls of high-quality libraries: 1—intact gDNA, 2—DNase I digestion of cross-linked chromatin under the conditions described by Ramani et al., and 3—ligation of DNase I-digested chromatin from lane 2

Our initial attempts to digest cross-linked chromatin using DNAse I showed that this step was not easy to reproduce. The distribution of fragment lengths obtained after digestion varied from sample to sample (Fig. 2a). Moreover, even at high enzyme concentrations, when the median fragment length was approximately 100 bp, a detectable amount of undigested high-molecular weight DNA was present in the reaction (as shown in Fig. 2a, lanes 4 and 5). Notably, our results showed that high-quality Hi-C libraries could not be obtained in this case (see the metrics of Protocol: Ma et al. in Fig. 1).

### Solution

Hi-C protocols include a nuclear permeabilization step, which allows subsequent enzymatic digestion of chromatin. This digestion is typically achieved by an SDS treatment in the presence of EDTA. However, DNase I is highly sensitive to the presence of metal chelators and SDS. To allow DNase I digestion after the permeabilization step, Ma et al. [22] suggested removing SDS and EDTA by sedimenting and washing chromatin. Alternatively, the DNase Hi-C protocol published by Ramani and colleagues [21] suggested not using EDTA and sequestering SDS with Triton X-100, similar to many classical Hi-C protocols. We aimed to compare the protocols of Ma et al., Ramani et al. and their modifications. As shown in Figs. 1a and 2a, in almost all cases, the protocol suggested by Ramani et al. resulted in:

1. A more reproducible DNase I digestion pattern with fragment lengths distributed between 100 and 1000 bp (a representative example is shown in Fig. 2 B, lane 2);
2. Higher overall protocol yields, and
3. A significantly lower noise ratio (measured as the percentage of *cis*-interactions).

We noted that the yield of DNA after biotin pulldown was a very good indicator of library quality. For example, libraries obtained without SDS quenching required more cycles of amplification after pulldown and resulted in a lower yield. In addition, analysis of sequencing reads with *FastContext,* a bioinformatic tool that we developed to assist with DNase Hi-C data analysis, showed that a large portion of reads from these low-quality libraries did not contain biotinylated adapters (Additional file 1: Fig. 2A), whereas the percentage of adapter-containing reads was higher for high-quality libraries (Additional file 1: Fig. S2B). Thus, libraries obtained without SDS quenching mostly represented undigested or unligated DNA.

### Quality controls

1. Always check digestion and ligation of the chromatin using gel electrophoresis. Representative results are shown in Fig. 2b, lanes 2–3.
2. Estimate the streptavidin pulldown efficiency and compare the pulldown yield with the unlabelled DNA control. Streptavidin pulldown of 1 µg of biotin-unlabelled genomic DNA followed by 12 cycles of PCR yielded ~ 30–42 ng (average = 36.2 ng, *n* = 4) of product. In contrast, we obtained at least 500 ng DNA from 6 cycles of PCR for successful library generation. This pulldown yield was similar when biotin labelling was performed using ligated biotinylated adapters or nucleotide exchange (see below). We found that it was critical to obtain several times more product after pulldown of libraries than in control reactions performed with the same amount of unlabelled DNA.

## Hint 2. Do not use unnecessary ingredients: excluding biotinylated adapters simplifies library preparation and data analysis

### Problem

In classical Hi-C and Micro-C protocols, the end-repair reaction with biotinylated nucleotides follows a digestion step, which allows DNA end-labelling and subsequent selection of ligation junctions. In the published DNAse Hi-C protocols, end-labelling is achieved via ligation of biotinylated oligonucleotides (adapters). Because ligation of adapters requires a sticky A-end, this reaction depends on the efficiency of A-tailing. In the recently published protocol by Ma et al., the authors additionally use a blunt adapter ligated to DNA ends if these ends that skip A-tailing. Adapter ligation introduces extra steps in the protocol, making it more complicated and less efficient.

**Fig. 3:**
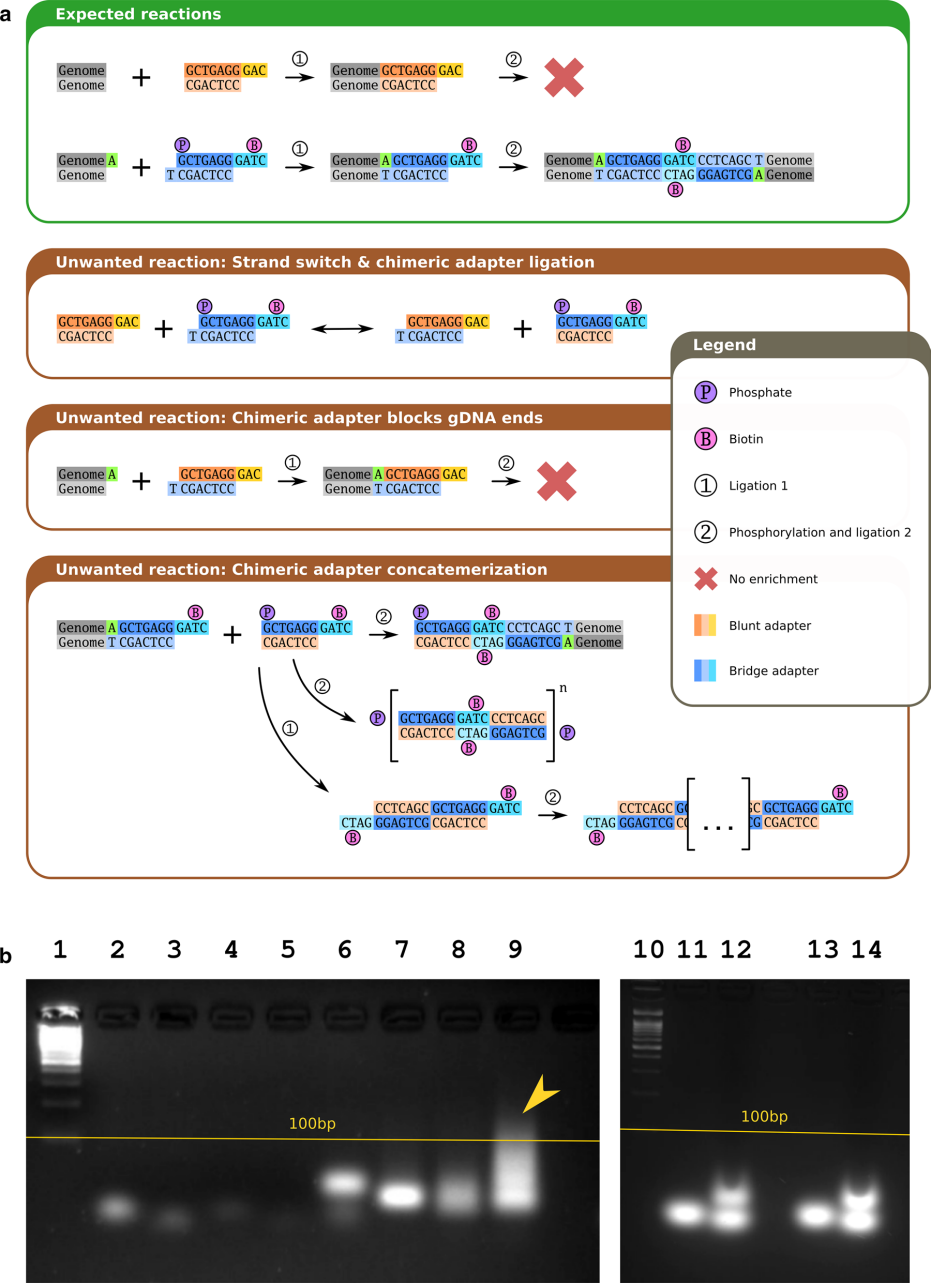
Using biotinylated adapters complicates the DNAse Hi-C protocol and results in undesired by-products. **a** Schematic description of possible ligation reaction products obtained in the presence of biotinylated adapters from Ma et al. [22]. The adapters used by Ma et al. are referred to as bridge and blunt oligonucleotides. As evident from the scheme, sequence similarity between bridge and blunt adapters leads to the formation of multimers. **b** Ligation assays showing the formation of adapter multimers. Lanes 1 and 10–100-bp ladder. Lanes 2 and 3—bridge (2) and blunt (3) oligonucleotides. Lanes 4–6—self-ligation assays performed with bridge (4), blunt (5), and bridge/blunt oligonucleotides mix (6). Lanes 7–9—bridge (7), blunt (8), and bridge/blunt oligonucleotides mix (9) subjected to ligation, followed by phosphorylation and an additional round of ligation, which imitated enzymatic steps during the DNAse Hi-C protocol. The arrow shows the adapter multimerization products. Lane 11—self-ligation assay of the redesigned blunt adapter, which lacks sequence similarity with the bridge adapter. Lane 12—redesigned blunt adapter subjected to ligation, phosphorylation and an additional round of ligation. Lanes 13 and 14—the same reactions as in lanes 11 and 12 performed using a mix of bridge and redesigned blunt-adapter oligonucleotides

Analysis of the data produced by Ma et al., as well as our own data, showed that oligonucleotide adapters were not only ligated to the DNA ends but also to each other, forming dimers and multimers (highlighted in yellow in Additional file 1: Fig. S2a). We illustrate in Fig. 3a that these undesired ligations between adapters can block proximity ligation, thereby leading to the low overall efficiency of the protocol and the increase of dangling ends and spurious interactions. Moreover, ligation of adapters made Hi-C data analysis more complicated, requiring the detection of adapter sequences and various ligation products between adapters in Hi-C reads before mapping (this problem will be additionally discussed below).

### Solution

We showed that the formation of adapter multimers was due to the sequence similarity of the blunt and bridge adapters (Fig. 3a) and could be reproduced in a control ligation reaction (Fig. 3b)*.* Changing the adapter sequences prevented the formation of adapter multimers (Fig. 3b), which led to the reduction of undigested/unligated fragments (see the corrected DEnds fraction metrics in Fig. 1a for the protocol “Ma et al., new blunt”). Additionally, we showed that the single adapter derived from the BAT-Hi-C protocol could be used as a substitute for the two-part bridge oligonucleotides. Using the single adapter allowed the generation of high-quality libraries (see the metrics in Fig. 1, “Protocol: Ramani et al., long linker” and “Protocol: Ma et al., long linker”) without using blunt adapters and a two-step ligation procedure, simplifying the experimental and computational steps.

We next decided to avoid the usage of any biotinylated oligonucleotide adapters. To achieve this aim, we performed end-labelling with biotinylated dCTP nucleotides during the end-repair step following DNase treatment. To the best of our knowledge, this report describes the first time the DNase Hi-C protocol has been performed without biotinylated adapters. We found that this simplified protocol allowed us to generate high-quality data (Fig. 1a, “Protocol: Ramani et al. + biotin fill-in”). Thus, we recommend using no adapters and employing a biotin fill-in strategy to prepare DNase Hi-C libraries.

### Quality control

1. If adapters were used for end-labelling, then a ligation assay was performed to ensure that the adapters could not form multimers during library preparation. Representative results are shown in Fig. 3b.
2. When processing data, we recommend searching for adapter multimers in read sequences. To achieve this aim, we developed the bioinformatic tool *FastContext*, which reports the relative abundance of different adapters and their combinations in sequenced reads. Representative results are shown in Additional file 1: Fig. S2.

## Hint 3. Do not treat dangling ends as artefacts

### Problem

We observed a large number of “dangling ends” fragments in the DNase Hi-C libraries. We quantified dangling ends as excess reads in the inward (forward–reverse, FR) orientation over reads in the same (forward–forward, FF or reverse–reverse, RR) orientation. This excess showed strong dependence on distance and sharply dropped when the distance between reads exceeded 1 kb. Excess FR reads are found in virtually all Hi-C libraries, and such reads are usually interpreted as representations of undigested or unligated DNA.

### Solution

We hypothesized that the large excess of FR reads might be due to frequent back-ligation events when the DNA ends were preferentially joined in the same order as in the intact (undigested) genome during the ligation step. We were able to assess the frequency of back ligations by analysing DNase Hi-C libraries prepared in the presence of biotinylated oligonucleotide adapters. The adapters marked ligation junctions; therefore, all reads in the FR orientation harbouring the adapter represented back-ligation events, rather than undigested or unligated chromatin. However, not all ligation junctions incorporated bridge adapters, some DNA ends could be ligated directly. To correctly account for this factor, we estimated the probability of adapter-free ligation events from the number of adapter-free interchromosomal read pairs, which represent all ligation events (“Methods” section). This approach allowed us to compute, for the first time, the frequency of back-ligation events in Hi-C libraries.

Notably, up to 75% of the excess FR reads were explained by back-ligation events (Fig. 1a; Additional file 1: Table S1). This result suggests that after digestion, DNA ends were preferentially located in close proximity to each other, which promoted back-ligation.

### Quality control

The high fraction of dangling ends does not necessarily reflect low digestion/ligation efficiency and should not be used as a quality control. Instead, the ratio of intra- to interchromosomal interactions reflects library quality. This metric can be computed using the computational tools described in the manuscript or other software [26].

## Hint 4. Burrows-Wheeler Aligner allows the analysis of Hi-C data without knowing the ligation junction motif

### Problem

**Fig. 4:**
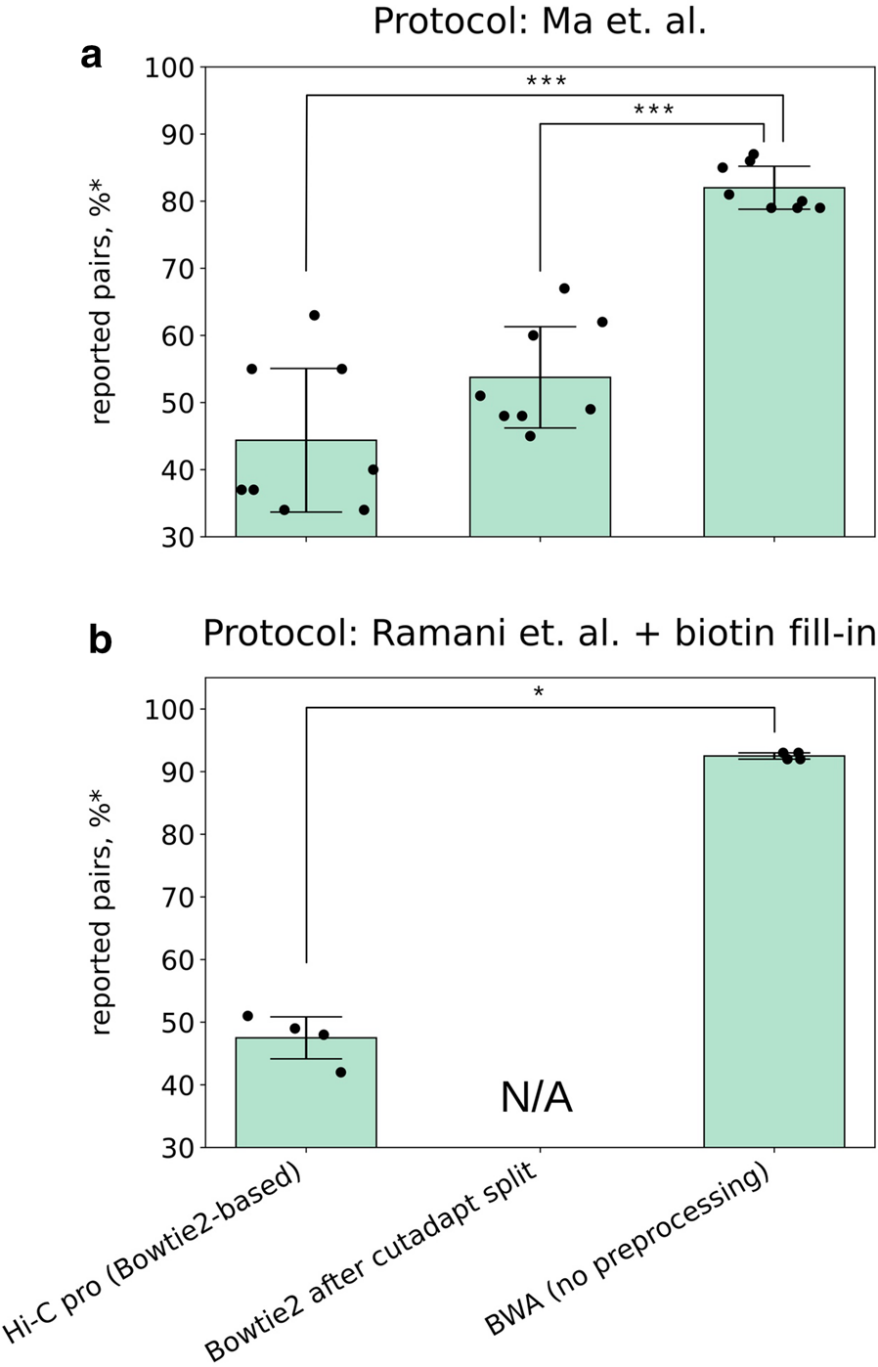
Burrows-Wheeler Aligner allows efficient mapping of Hi-C reads both in the presence and in the absence of adapter sequences. For samples prepared using protocol Ma et al. (**a**), end-labelling was achieved by adapter ligation, whereas for samples prepared using protocol Ramani et al., biotin fill-in (**b**) DNA, we did not use any internal adapters. Reads were mapped by Hi-C pro [28] (bowtie2-based software) directly or, in the case of protocol Ma et al., after removing the adapter sequences (cutadapt split). Alternatively, reads were aligned using bwa without any pre-processing. Mapping efficiency is shown as a percentage of alignable reads (reported pairs). Each dot represents an independent Hi-C library preparation, and the significance of differences was computed using the Mann–Whitney test

Many Hi-C reads are chimaeric, i.e. they contain junctions between different genomic fragments. This feature might affect the mappability of Hi-C reads. When using restriction enzymes to digest chromatin, a specific ligation junction site allows chimaeric read splitting. A similar approach can be employed when using bridge adapters in the DNAse Hi-C protocol. However, we observed that using adapters reduced Hi-C read mappability (as evident from the number of reported pairs in Fig. 1a, Additional file 1: Table S1, and Fig. 4a and b), and splitting chimaeric reads containing expected adapter junction sequences could not fully address this problem (Additional file 1: Table S2, Fig. 4a). Analysis of unaligned reads showed that they contained adapter multimers, both at the read ends and in the middle of the fragments. This finding was in line with our observations of adapter multimerization. Removing these adapter sequences improved the alignment (Fig. 4a), but required sophisticated bioinformatic pipelines (“Methods” section). Finally, for libraries prepared without bridge adapters, as suggested above, it is impossible to find ligation junctions before alignment.

### Solution

We found that Burrows-Wheeler Aligner (bwa) [27] could efficiently align chimaeric reads (Fig. 4b). For reads containing adapter multimers, reads split (or trimmed) using expected adapter sequences, and reads obtained from DNAse Hi-C libraries without adapters, bwa showed significantly better results than another commonly used aligner, bowtie2 (Additional file 1: Table S2, Fig. 4b). Thus, we recommend using bwa for Hi-C data processing and note that the adapter trimming step is not necessary.

### Quality control

For human data, approximately 90% of pairs could be mapped unambiguously at both ends when using bwa for mapping. The mapping statistics can be accessed using the pipeline accompanying the manuscript.

## Discussion

DNAse Hi-C methods are used relatively rarely, most likely due to their complicated experimental design, which includes optimization of the chromatin digestion conditions and the use of biotinylated adapters. Our results showed that the DNAse I digestion step could be reproducible when using optimal cell lysis and SDS quenching conditions, as suggested in [21]. Furthermore, we showed that the use of biotinylated adapters was not necessary and that biotin could be incorporated by applying a fill-in strategy. Although the fill-in and adapter-based strategies both resulted in high-quality data when using optimal cell lysis and SDS quenching conditions, the former simplified the experimental protocol and prevented the formation of ligation by-products.

Finally, there was no need to identify the ligation junction sites within sequencing fragments because Burrows-Wheeler Aligner could efficiently map chimaeric reads. Overall, our work shows that DNAse I Hi-C is a robust and efficient method that can be easily applied to study chromatin interactions in human cells.

Notably, we found that using a single biotinylated nucleotide (biotin-dCTP in our case) was sufficient for labelling DNA ends. This could be explained by several observations. First, DNAse I in the presence of Mn2 + introduces double-stranded breaks with 5′-overhangs with a length of 4 bp [32, 33]. When filling this overhang, biotin-dCTP nucleotides are likely to be introduced.

Second, even if a fraction of DNA ends appear to be blunt, the Klenow enzyme has 3′-5′ exonuclease activity, which enables the exchange of nucleotides at blunt ends. A previous study [34] suggests that not only terminal, but also internal nucleotides can be labelled due to this activity, which reduces the dependence of labelling on the sequence of the DNA end.

Finally, because both blunt- and cohesive-ends could be labelled and DNA breaks occur independently in each cell, it follows that labelling with biotinylated dCTP (or any other single biotinylated dNTP) could occur in almost any genomic region.

The main advantage of the DNAse I Hi-C method is that it enables more uniform coverage to be achieved than the conventional Hi-C technique. However, we note that there are other approaches aimed at generating Hi-C libraries with uniform coverage employing, for example, MNase to fragment the genome [24]. Moreover, DNase and MNase have their own smaller and larger sequence-specific biases, respectively, and using a combination of several 4-cutter restriction endonucleases (up to three in the Arima Genomics kit) could provide comparable, if not the same, coverage as DNAse I.

Analysis of the ligation junctions marked by biotinylated oligonucleotides showed that the orientation of DNA ends during the ligation step was not random. Our data demonstrated a strong preference for the ligation of DNA fragments in the same order as they occur in the intact genome. This preference is most likely due to tight formaldehyde fixation, which does not allow rotation or diffusion of DNA ends after digestion. Interestingly, the number of excess FR reads varies from library to library, which probably reflects differences in the level of chromatin fixation. Thus, excess FR reads should not be used to score the quality of Hi-C libraries.

## Conclusions

By comparing and modifying existing methods, we developed a robust and efficient protocol for DNAse I Hi-C analysis of human cells and tissues. We demonstrated the reproducibility of this protocol by applying it to human blood samples. The end-labelling strategy employed in the protocol does not require the incorporation of biotinylated adapters, and DNAse I digestion results in more uniform coverage than restriction enzyme-based approaches. Uniform coverage and the absence of exogenous sequences, which could be erroneously aligned to the reference genome, make this protocol suitable for SNP detection. In addition, the lower noise levels of the protocol developed in this study compared to previously published DNAse I Hi-C protocols should be beneficial for studying the 3D organization of chromatin and detecting chromosomal rearrangements. Thus, the protocol developed in this study could be used in the future to characterize genetic polymorphisms and study chromatin architecture in human cells.

## Methods

### Detailed DNase Hi-C protocols

We describe the protocol “Ramani et al., biotin fill-in”. This is the main protocol developed in this study, which allows the preparation of high-quality Hi-C libraries without biotinylated adapters. The additional protocols used in this study are described in the Additional file 1.

1. Cell crosslinking
  1.1. Preparation of cell suspensions
    A. Peripheral blood samples
      Peripheral blood samples were collected in tubes with EDTA. RBC lysis buffer (BioLegend) was used for erythrocyte removal according to the manufacturer’s instructions. After lysis, the cell pellets were washed, cells were counted and resuspended in DMEM at a concentration of 1 × 10^6^ cells/ml.
    B. Suspension cells
      K562 cells were grown in RPMI-1640 medium with 10% FBS and a penicillin/streptomycin mix (all from Invitrogen). The cells were collected, washed to remove traces of serum and resuspended in RPMI-1640 medium at the same concentration.
    C. Adherent cells
      Adherent LNCap and A549 cells were grown in DMEM with 10% FBS and a penicillin/streptomycin mix (all from Invitrogen). The cells were disaggregated by a trypsin (Invitrogen) treatment, washed and resuspended in DMEM at the same concentration.
  1.2. Cell fixation
    For all cell types, cells were fixed by adding 1% formaldehyde (Sigma-Aldrich) and incubating for 10 min at room temperature (RT) with continuous rotation. Crosslinking was quenched by adding 2.5 M glycine to a final concentration of 0.125 mM and incubating for 10 min at RT with continuous rotation. The cell suspension was centrifuged at 1100*g* for 10 min, resuspended in PBS and split into aliquots of 2.5 × 10^6^ cells. The cells were centrifuged at 1100*g* for another 10 min. The cell pellets were snap-frozen and stored at  −  80 °C.
2. Cell lysis
  2.1. The pellet of cross-linked cells was placed on ice and gently resuspended in 1 ml of ice-cold cell lysis buffer (10 mM Tris–HCl pH 8.0, 10 mM NaCl, 0.2% Igepal).
  2.2. The pellet was then incubated on ice for 20 min with intermittent rotation.
  2.3. Centrifugation was performed at 2500*g* for 5 min.
  2.4. The supernatant was removed, and the pellet was gently resuspended in 100 μl DNase buffer (50 mM Tris–HCl pH 7.5, 0.5 mM CaCl_2_) with 5 mM MnCl_2_ and 0.2% SDS.
  2.5. The resuspended pellet was incubated at 37 °C for 30 min.
    Control point 1: 5 μl lysed cells were saved to check the DNase I digestion efficiency.
  2.6. SDS was quenched by adding 100 μl DNase buffer (50 mM Tris–HCl pH 7.5, 0.5 mM CaCl_2_) with 5 mM MnCl_2_ and 2% Triton X-100
  2.7. The quenched reaction mixture was incubated at 37 °C for 10 min.
  2.8. DNase I (1.5 U; Thermo Scientific) was added and incubated at RT for 5 min (note: do not incubate mixture longer than 5 min).
  2.9. The reaction was stopped immediately after 5 min by adding 40 μl Stop buffer (125 mM EDTA, 2.5% SDS).
    Control point 2: 10 μl of the reaction was saved to check the DNase I digestion efficiency. Ninety microlitres of lysis buffer (10 mM Tris–HCl pH 8.0, 10 mM NaCl, 0.2% SDS) and 5 μl proteinase K (800 units/ml) were added to both controls. The controls were reverse cross-linked at 65 °C for at least 3 h. DNA was extracted by the standard phenol–chloroform method.
  2.10. The reaction was centrifuged at 2500*g* for 5 min.
  2.11. The supernatant was discarded, and the pellet was resuspended in 100 μl nuclease-free water.
  2.12. Two hundred microlitres of AMPure beads were added and mixed well.
  2.13. The mixture was incubated for 5 min at RT, and the tube was exposed to a magnet for 2 min.
  2.14. The supernatant was discarded, and the beads were washed twice with freshly prepared 80% ethanol. The mixture was spun gently (no more than 2500*g*), and residual ethanol was removed. The beads were air dried for no more than 2 min.
  2.15. The beads were resuspended in 100 μl NEBuffer 3.1, and the AMPure Beads remained in the mixture.
3. Biotin labelling (volume: 200 μl)
  3.1. The following components were mixed on ice:
    **Table:** 

Reagents	Amount per tube (μl)	Final
NEBuffer 3.1	10	1X
dATP, 10 mM	1.5	75 μM
dTTP, 10 mM	1.5	75 μM
dGTP, 10 mM	1.5	75 μM
Biotin-15-dCTP, 1 mM	15	75 μM
Klenow (5 U/μl)	10	50U
H_2_O	60.5
  3.2. The above components were added to the tube from step 2.14.
  3.3. The mixture was incubated at 23 °C on a thermomixer for 4 h with intermittent gentle shaking.
4. In situ ligation (volume 1000 μl)
  4.1. The following components were mixed on ice:
    **Table:** 

Reagents	Amount per tube (μl)	Final
10 × T4 ligase buffer	100	1X
10% Triton X-100	100	1%
25% PEG-8000	200	5%
BSA 100 mM	10	1 mM
T4 DNA ligase ()	20	
H_2_O	370
  4.2. The above components were added to the tube from step 3.3.
  4.3. The mixture was incubated at 16 °C on a thermomixer for at least 8 h (night is also appropriate) with continuous shaking.
5. Cross-link reversal
  5.1. Centrifuge the reaction mixture obtained after step 4.3 at 2500*g* for 3 min.
  5.2. The supernatant was discarded, and the pellet was resuspended in 400 μl NEBuffer 2.
  5.3. Twelve microlitres of 10% SDS was added to the resuspended pellet.
  5.4. Twenty microlitres of proteinase K (800 units/ml) was then added to the mixture.
  5.5. The mixture was incubated at 65 °C on a thermomixer for 4 h with continuous shaking.
  5.6. Twenty microlitres of proteinase K (800 units/ml) was then added.
  5.7. The mixture was incubated at 65 °C on a thermomixer for 4 h (this step can be performed at night) with continuous shaking.
  5.8. Three microlitres Glycoblue, 50.5 μl 3 M NaAc and 506 μl isopropanol were then added.
  5.9. The mixture was incubated at − 80 °C for 20 min.
  5.10. The mixture was then centrifuged at greater than 15,000*g* for 40 min at 4 °C.
  5.11. The supernatant was discarded, and the DNA and AMPure Beads pellet was resuspended with 100 μl nuclease-free water containing 5 μg RNase A.
  5.12. The resuspended pellet was incubated at 37 °C on a thermomixer for 30 min with continuous shaking.
  5.13. Fifty microlitres AMPure beads (0.5x) were added and mixed well.
  5.14. The mixture was incubated for 5 min at RT, and the tube was exposed to a magnet for 2 min.
  5.15. The supernatant was discarded, and the beads were washed twice with freshly prepared 80% ethanol and spun briefly. The residual ethanol was removed. Then, the beads were air dried for no more than 2 min.
  5.16. The beads were resuspended in 50 μl nuclease-free water.
  5.17. The beads were incubated for 10 min at RT and then collected via a magnet. The supernatant was transferred to a new 1.5 ml tube.
  5.18. The concentration of the recovered DNA was measured with a Qubit fluorometer. The yield was 3–6 μg if starting with 2.5 × 10^6^ cells.
6. Removal of biotin from unligated ends (volume: 100 μl)
  6.1. The following components were mixed on ice:
    **Table:** 

Reagents	Amount per tube (μl)	Final
Purified DNA	50	
NEBuffer 2.1	10	1X
dATP, 1 mM	1.2	12 μM
dGTP, 1 mM	1.2	12 μM
EDTA, 25 mM	1	0.25 mM
T4 DNA polymerase (3U/μl)	1.6	5U
H_2_O	35
  6.2. The above components were incubated at 20 °C in a thermocycler for 1 h for 30 min.
    We strongly recommend testing T4 DNA polymerase before proceeding with the following steps.
  6.3. The reaction was stopped by adding 5 μl 500 mM EDTA.
  6.4. One hundred and five microlitres AMPure beads (1x) were added and mixed well.
  6.5. The mixture was incubated for 5 min at RT, and the tube was exposed to a magnet for 2 min.
  6.6. The supernatant was discarded, and the beads washed twice with freshly prepared 80% ethanol and spun briefly. The residual ethanol was removed. Then, the beads were air dried for no more than 2 min.
  6.7. The beads in were resuspended in 120 μl nuclease-free water.
  6.8. The beads were incubated for 10 min at RT and collected via a magnet. The supernatant was transferred to a new 1.5-ml tube.
  6.9. The concentration of the recovered DNA was measured with a Qubit fluorometer. It is normal if the amount of DNA is about 30–70% of original amount after these steps.
7. Sonication
  7.1. DNA was transferred to a Covaris microtube.
  7.2. DNA was sheared to a size of 200–400 bp. For Covaris, the following parameters were used: duty factor, 20; peak power, 50; cycles per burst, 200; and time, 110 s
  7.3. Sheared DNA was transferred to a new 1.5-ml tube marked as “L”.
8. Size select
  8.1. The volume of sheared DNA (tube “L”) was brought to 200 μl with nuclease-free water.
  8.2. One hundred and twenty-five microlitres of AMPure beads were added and mixed well.
  8.3. The mixture was incubated for 10 min at RT and exposed to a magnet for 2 min.
  8.4. Meanwhile, 200 μl AMPure beads were added to a new 1.5-ml tube (marked as “S”), and the tube was exposed to a magnet for 2 min.
  8.5. The supernatant was discarded, and 100 μl AMPure beads were added and mixed well.
  8.6. The supernatant from tube “L” was transferred to tube “S” and mixed well.
  8.7. The mixture was incubated for 10 min at RT, and the tube was exposed to a magnet for 2 min.
  8.8. The supernatant was discarded, and the beads were washed twice with freshly prepared 80% ethanol and spun briefly. Residual ethanol was removed. Then, the beads were air dried for no more than 2 min.
  8.9. The beads were resuspended in 50 μl nuclease-free water.
  8.10. The beards were incubated for 10 min at RT and collected via a magnet. The supernatant was transferred to a new 1.5-ml tube.
  8.11. The concentration of the recovered DNA was measured with a Qubit fluorometer. DNA amount could be about 50% of initial amount after these steps.
9. End repair and dA-tailing (volume 60 μl). We used the KAPA HyperPrep Kit.
  9.1. One microgram purified DNA was transferred to a new 0.2-ml tube.
  9.2. The volume of DNA was brought to 50 μl with nuclease-free water.
  9.3. The following components were mixed on ice:
    **Table:** 

Reagents	Amount per tube (μl)
End repair and A-tailing buffer	7
End repair and A-tailing enzyme mix	3
  9.4. The above components were added to the tube from step 9.1.
  9.5. The mixture was incubated at 20 °C for 30 min and then at 65 °C for 30 min in a thermocycler with the lid at 85 °C to inactivate enzymes.
10. Adapter ligation (volume: 110 μl). We used the KAPA HyperPrep Kit.
  10.1 The following components were mixed on ice:

**Table:** 

Reagents	Amount per tube (μl)
DNA from the step 9.5	60
H_2_O	5
Adapter stock (concentration as required)	5
Ligation buffer	30
DNA Ligase	10
  10.2. The above components were incubated at 20 °C for 30 min in a thermocycler with the lid OFF.
  10.3. Eighty-eight microlitres AMPure beads were added and mixed well.
  10.4. The mixture was incubated for 5 min at RT, and the tube was exposed to magnet for 2 min.
  10.5. The supernatant was discarded, and the beads were washed twice with freshly prepared 80% ethanol and spun briefly. Residual ethanol was removed. Then, the beads were air dried for no more than 2 min.
  10.6. The beads were resuspended in 100 μl nuclease-free water.
  10.7. The beads were incubated for 10 min at RT and were collected via a magnet. The supernatant was transferred to a new 1.5-ml tube.
11. Biotin pulldown
  11.1. Thirty microlitres Dynabeads® MyOne™ Streptavidin C1 and 100 μl of 1 × B&W buffer (5 mM Tris–HCl pH 8.0, 0.5 mM EDTA, 1 M NaCl) were mixed in a 1.5-ml low binding tube, and the tube was exposed to a magnet for 2 min.
  11.2. The supernatant was discarded, and 100 μl of 1 × B&W buffer was added to the beads and mixed well. The tube was exposed to a magnet for 2 min.
  11.3. The supernatant was discarded, and 100 μl 2 × B&W buffer (10 mM Tris–HCl pH 8.0, 1 mM EDTA, 2 M NaCl) was added.
  11.4. Purified adapter-ligated DNA was added to the beads from step 10.7 and mixed well.
  11.5. The mixture was incubated for 15 min at RT with rotation.
  11.6. The tube was exposed to a magnet for 2 min, and the supernatant was discarded.
  11.7. The beads were washed four times with 200 μl 1 × B&W buffer with the addition 0.1% Tween-20.
  11.8. The tube was washed two times with 200 μl 10 mM Tris–HCl, pH 8.0, and before the last wash, the tube was changed.
  11.9. The beads were resuspended in 40 μl 10 mM Tris–HCl, pH 8.0.
12. Amplification (volume 50 μl). We used the KAPA HyperPrep Kit.
  12.1. The following components were mixed on ice:

**Table:** 

Reagents	Amount per tube (μl)	Final
DNA-bound streptavidin beads	20	
KAPA HiFi HotStart ReadyMix (2x)	25	1x
KAPA Library Amplification Primer Mix (10x)	5	1x
  12.2. The following PCR program was used:
    1 cycle—Initial denaturation, 98 °C for 45 s—1 cycle
    0–20 cycle—Denaturation, 98 °C for 15 s.
    Annealing, 60 °C for 30 s.
    Extension, 72 °C for 30 s.
    1 cycle – Final extension, 72 °C for 1 min
    HOLD 4 °C ∞.
  12.3. Fifty microlitres AMPure beads were added to the reaction mix and mixed well.
  12.4. The mixture was incubated for 5 min at RT, and the tube was exposed to a magnet for 2 min.
  12.5. The supernatant was discarded, and the beads were washed twice with freshly prepared 80% ethanol and spin briefly. Residual ethanol was removed. Next, the beads were air dried for no more than 2 min.
  12.6. The beads were resuspended in 40 μl nuclease-free water.
  12.7. The beads were incubated for 10 min at RT and were collected via a magnet. The supernatant was transferred to a new 0.5-ml tube.
  12.8. The concentration of the recovered DNA was measured with a Qubit fluorometer.
13. Check the quality.
  13.1 Two nanograms of the amplified library was analysed using an Agilent High Sensitivity DNA Kit according to the manufacturer's instructions. The library displayed a fragment size distribution in the range of 150 to 500 bp.

## Computational analysis of DNase Hi-C libraries

### Mapping and processing of sequence reads

We sequenced the targeted DNase Hi-C libraries using paired-end reads with a length of 150 bp. Next, we mapped the paired-end reads to the human hg19 genome using BWA-MEM [27] with the default parameters. We did not remove the Illumina adapters before mapping and decided to not split the reads by ligation junctions because bwa could successfully map the reads (Additional file 1: Table S2, Fig. 3). To define interacting genomic fragments, we searched for the greatest distance between the coordinates of all the primary and supplementary alignments from both read mates. To obtain valid interaction pairs, we only included reads with both mates mapped uniquely (mapq > 0). We removed PCR duplicates from the Hi-C data: we defined two read pairs as duplicates if they shared the alignment position of both mates. Unique read filtering and duplicate removal were performed using an in-house developed pipeline. The scripts that we used for Hi-C data processing and quality analysis are available on GitHub https://github.com/evgeniy240294/ExoC.

As an alternative to bwa, we used bowtie2 [29] wrapped in the Hi-C Pro [28] pipeline. We used Hi-C Pro with the default parameters. We provided the sequence of the bridge adapter as a ligation junction sequence. To remove the adapter sequences from the read ends, we used cutadapt [30] iteratively in the noninternal adapter mode with the following parameters: minimum overlap equal to 5 and minimum length equal to 7. We searched for all the variants of the multimers in every iteration. We performed five consecutive iterations of cutadapt processing to remove adapter concatemers.

### Quality control of Hi-C data

To evaluate the number of dangling ends ($\text{DE}$), we used the following equation:

$$\text{DE}=(FR+RF)-(FF+RR),$$

where $FR, RF, FF, \, \text{and} RR$ are the number of valid pairs with read mates in the forward–reverse, reverse–forward, forward–forward and reverse–reverse orientations, respectively. It was assumed that the $FR, RF, FF \text{and} RR$ classes of the Hi-C read orientations were distributed at a ratio of 1:1:1:1 and that overrepresentations of $FR$ or $RF$ might indicate the presence of either nonligated fragments or back ligations, respectively.

To evaluate the number of back ligations ($B$) in $\text{DE}$, we used datasets prepared using a biotinylated oligonucleotide adapter. This allowed the identification of a fraction of reads that had bridge adapters in their sequence. First, we used cutadapt as described above to remove adapters from the 5′-ends of reads, thus keeping only reads containing noninternal adapter sequences. Notably, the read length was 150 bp; therefore, the adapter sequence could be undetected if the DNA insert was more than 300 bp. Therefore, we only considered reads that were less than 300 bp. For this purpose, we used the AdapterRemoval tool [31] without specifying any adapter sequence and with the default parameters. In this mode, the tool allowed us to find all the reads that had two mate sequences overlapping with each other, which means that they represented DNA fragments with inserts of less than 300 bp completely covered by two mates with a length of 150 bp.

The presence of an adapter within a read sequence could indicate either that the adapter was ligated to the DNA end during Hi-C library preparation or that the genomic sequence matched the adapter sequence by chance. Thus, if we considered all the read pairs in the FF or RR orientation containing adapter sequences, we could describe them as the sum of adapter ligation events and incidental matches between the adapter sequence and genomic DNA. Formally, we define:

$$P1=N\cdot \left(y+c\right),$$

where P1 is the number of reads in the FF and RR orientations containing the adapter sequence, N is the total number of FF and RR reads, *y* is the frequency of adapter ligation and *c* is the frequency of the incidental occurrence of the adapter sequence within the genome. To compute *c,* we searched for the adapter sequences in the libraries prepared without an adapter (in this case, *y *= 0) and found that $c\approx 0.05$ for the 7-bp bridge adapter and $c\approx 0$ for a 19-bp long BAT-Hi-C adapter.

Next, we considered the reads in the FR and RF orientations containing the adapter sequence. Without preference for back-ligation and in the absence of undigested DNA, the number of such reads would be similar to the number of FF and RR reads, which is equal to $N\cdot (y+c)$. The back-ligation events expected for equal FF/RR/RF/RR orientations would add to this number $B\cdot (y + c)$, where $B$ is the number of back-ligation events. Reads originating from undigested/unligated DNA could contain the adapter sequence only when it incidentally matched the genomic sequence, which would add $G\cdot c$ reads in the FR or RF orientation with adapter, where $G$ is the number of sequenced undigested/unligated DNA fragments. In total, the number of reads in the FR and RF orientations containing the adapter sequence, which we defined as P2, can be defined as

$$P2=N\cdot \left(y+c\right)+G\cdot c+B\cdot \left(y + c\right)=N\cdot \left(y+c\right)+\left(G+B\right)\cdot c + B\cdot y.$$

In this equation, it is pertinent to note that $(G+B)$ is the sum of back-ligation events and undigested/unligated DNA, which we have previously estimated as $\text{DE}$, i.e. $(G+B)=\text{DE}$. We also know that $P1=N\cdot (y+c)$; therefore,

$$P2=P1+\text{DE} \cdot c +B \cdot y$$, and

$$y=\frac{P1}{N}-c.$$

This allows us to compute $B$ as follows:

$$B=\frac{P2-P1-DE\cdot c }{\frac{P1}{N}-c}.$$

Using this equation, we computed *B* for all the libraries prepared with biotinylated oligonucleotide adapters.

## Supplementary Information

**Additional file 1.** Supplementary information: supplementary tables and figures.

## Data Availability

Sequencing data are accessible via the NCBI GEO database. The scripts used for data processing and analysis are available on GitHub: https://github.com/evgeniy240294/ExoC and https://github.com/regnveig/labjournal/tree/master/tools/FastContext.
