# Supplementary material for: A cookbook for DNase Hi-C
Source: Epigenetics Chromatin. 2021 Mar 20;14:15. doi: 10.1186/s13072-021-00389-5 (PMC7981840; doi:10.1186/s13072-021-00389-5)
Supplement: Supplementary file 1 — Additional file 1. Supplementary information: supplementary tables and figures. [file 13072_2021_389_MOESM1_ESM.pdf]

Supplementary Figure 1. Representative Hi-C maps for DNAse I Hi-C protocols

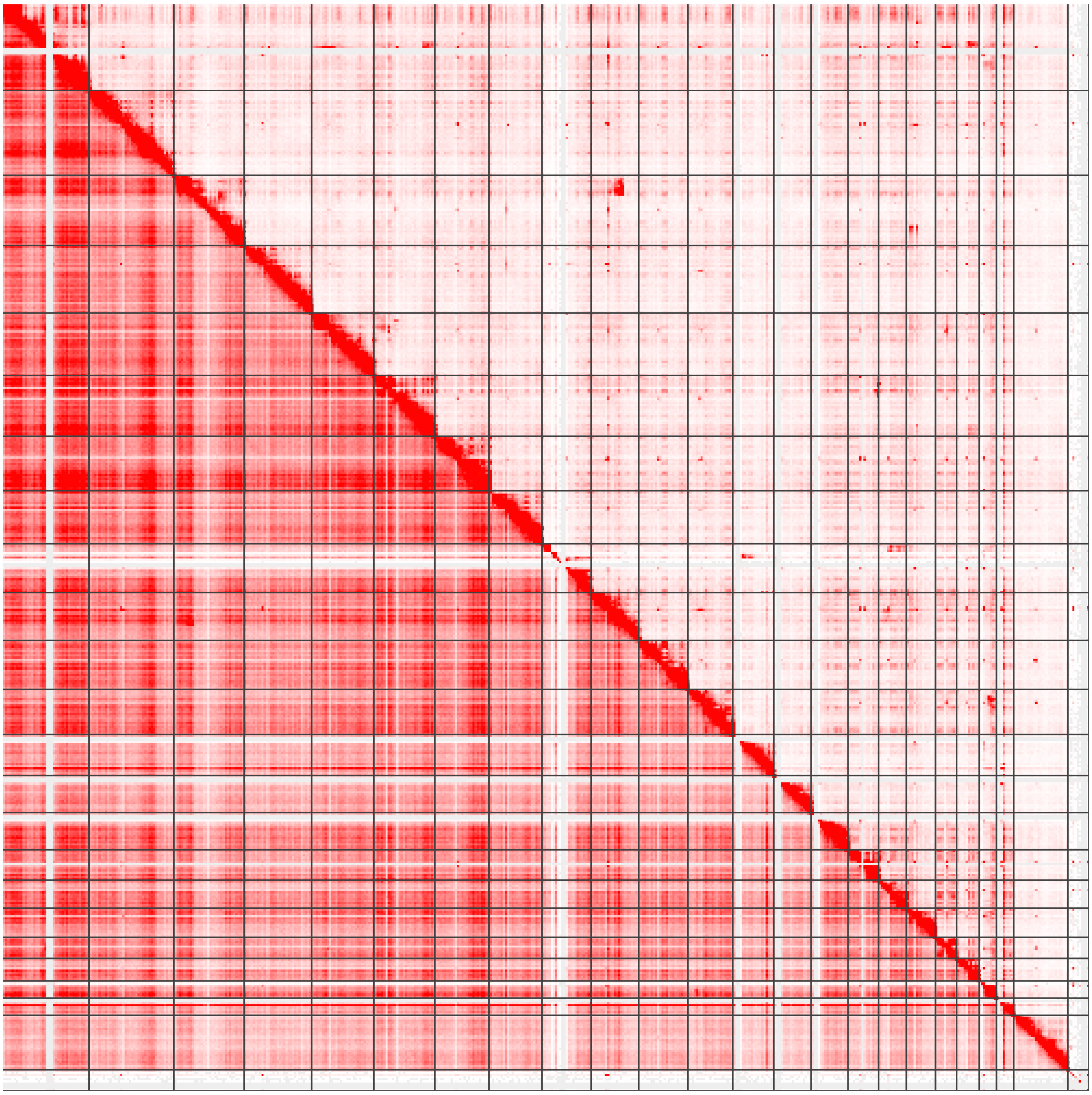

Above diagonal: Protocol Ramani et al., biotin fill-in
Below diagonal: Ma et al. (reanalyzed), K562, capture
Full-genome view
Color scale: 535

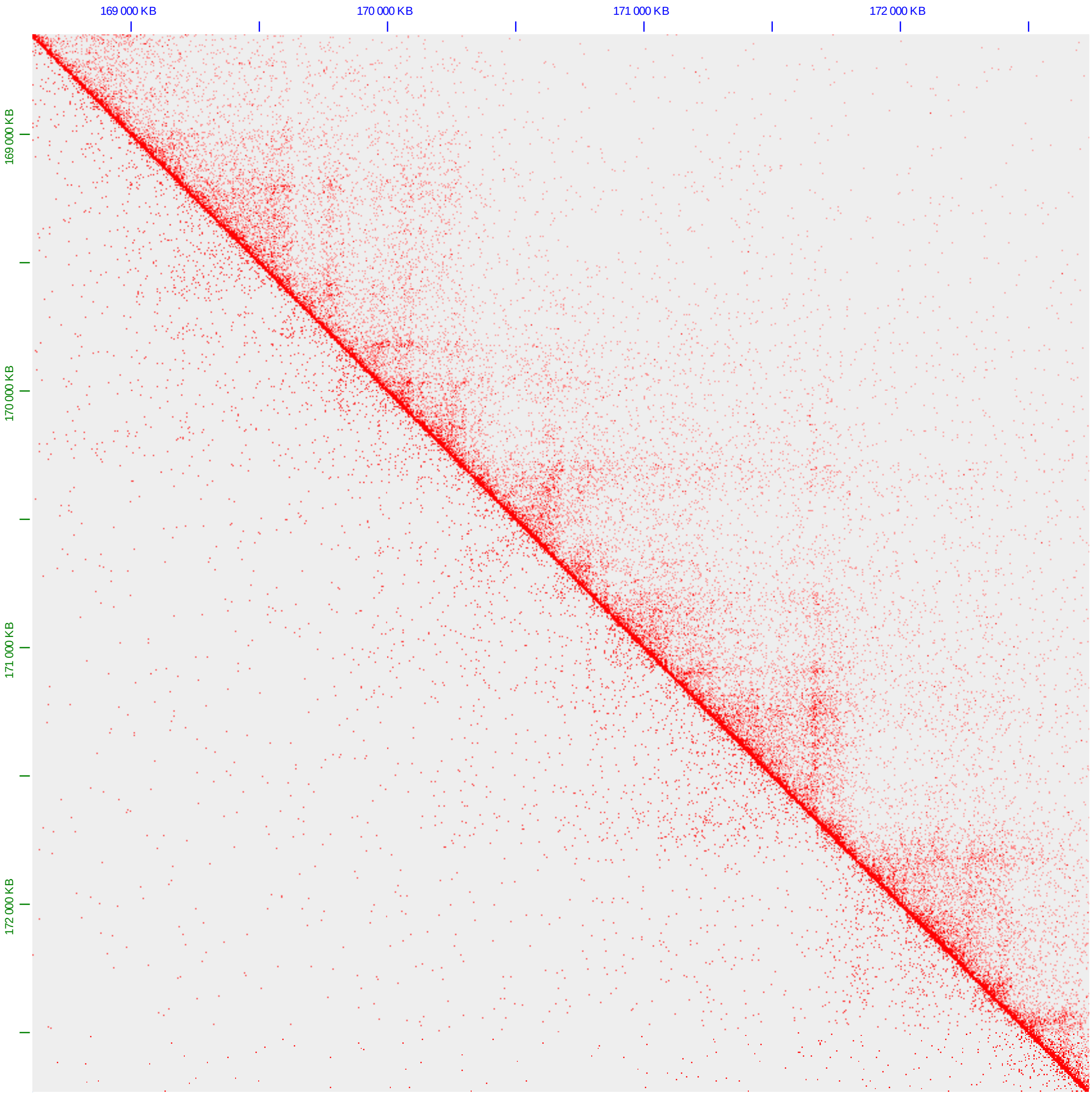

Above diagonal: Protocol Ramani et al., biotin fill-in
Below diagonal: Ma et al. (reanalyzed), K562, capture
Resolution: 5kb
Chromosome: chr2
Color scale: 1

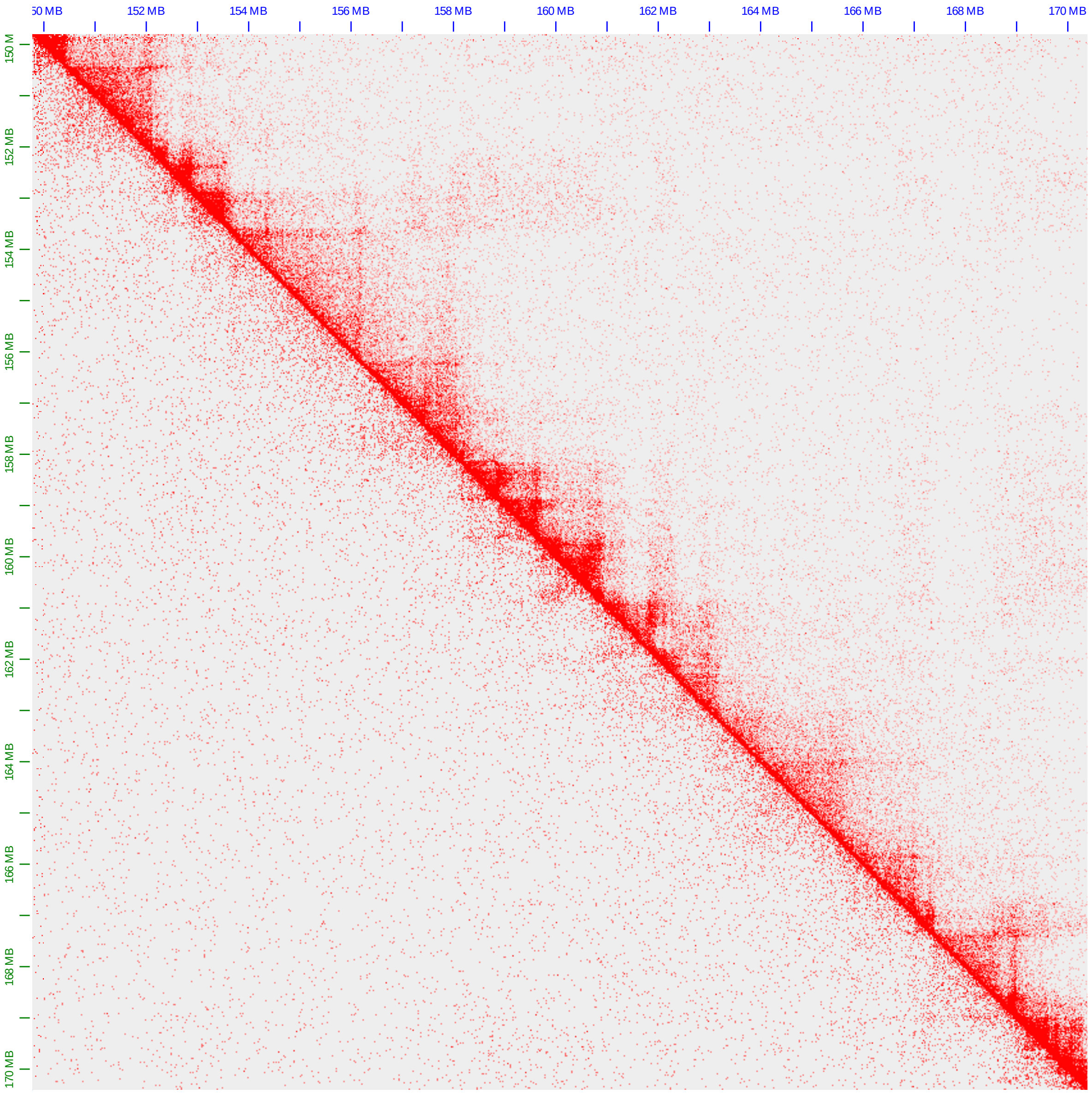

Above diagonal: Protocol Ramani et al., biotin fill-in
Below diagonal: Ma et al. (reanalyzed), K562, capture
Resolution: 25kb
Chromosome: chr2
Color scale: 2

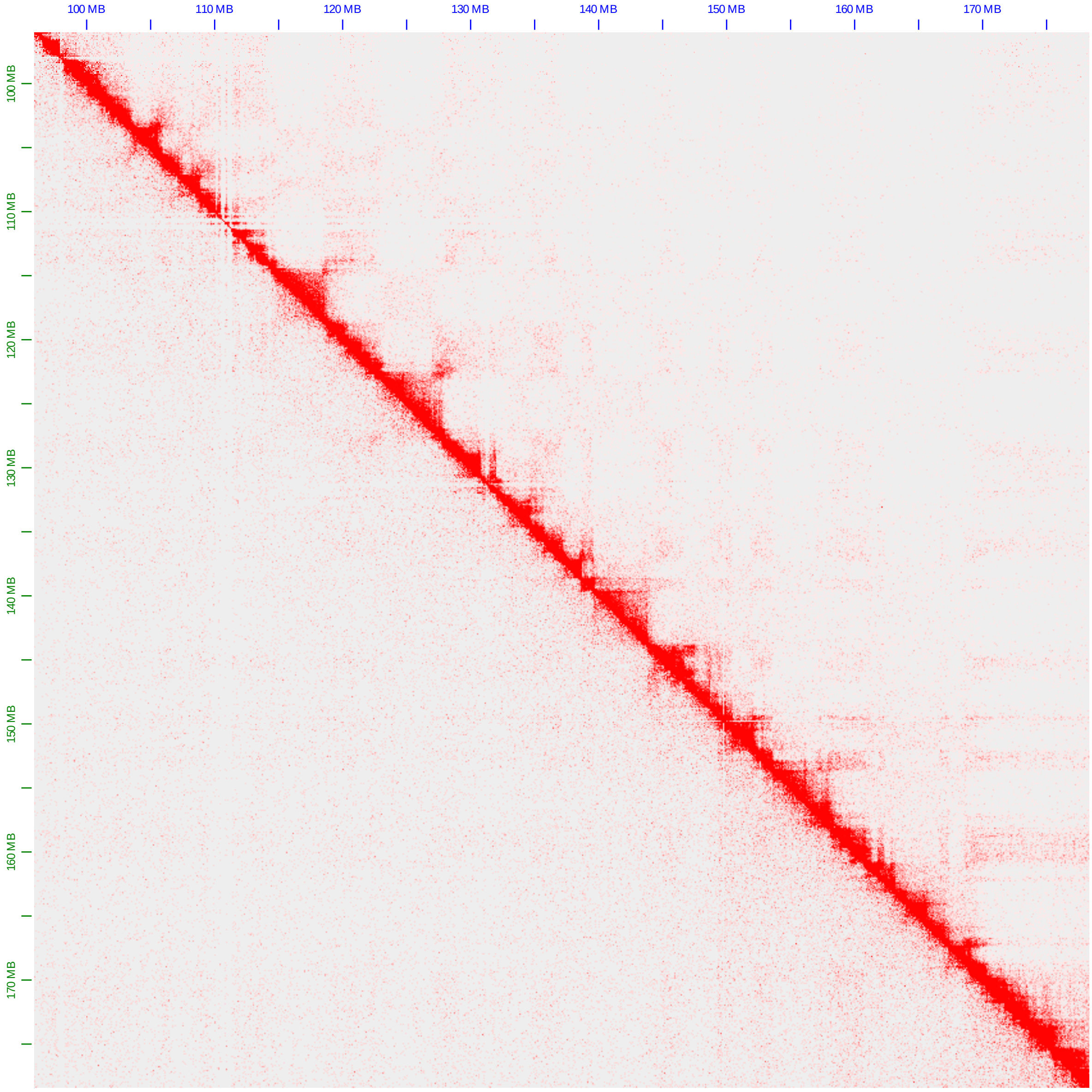

Above diagonal: Protocol Ramani et al., biotin fill-in
Below diagonal: Ma et al. (reanalyzed), K562, capture
Resolution: 100kb
Chromosome: chr2
Color scale: 8

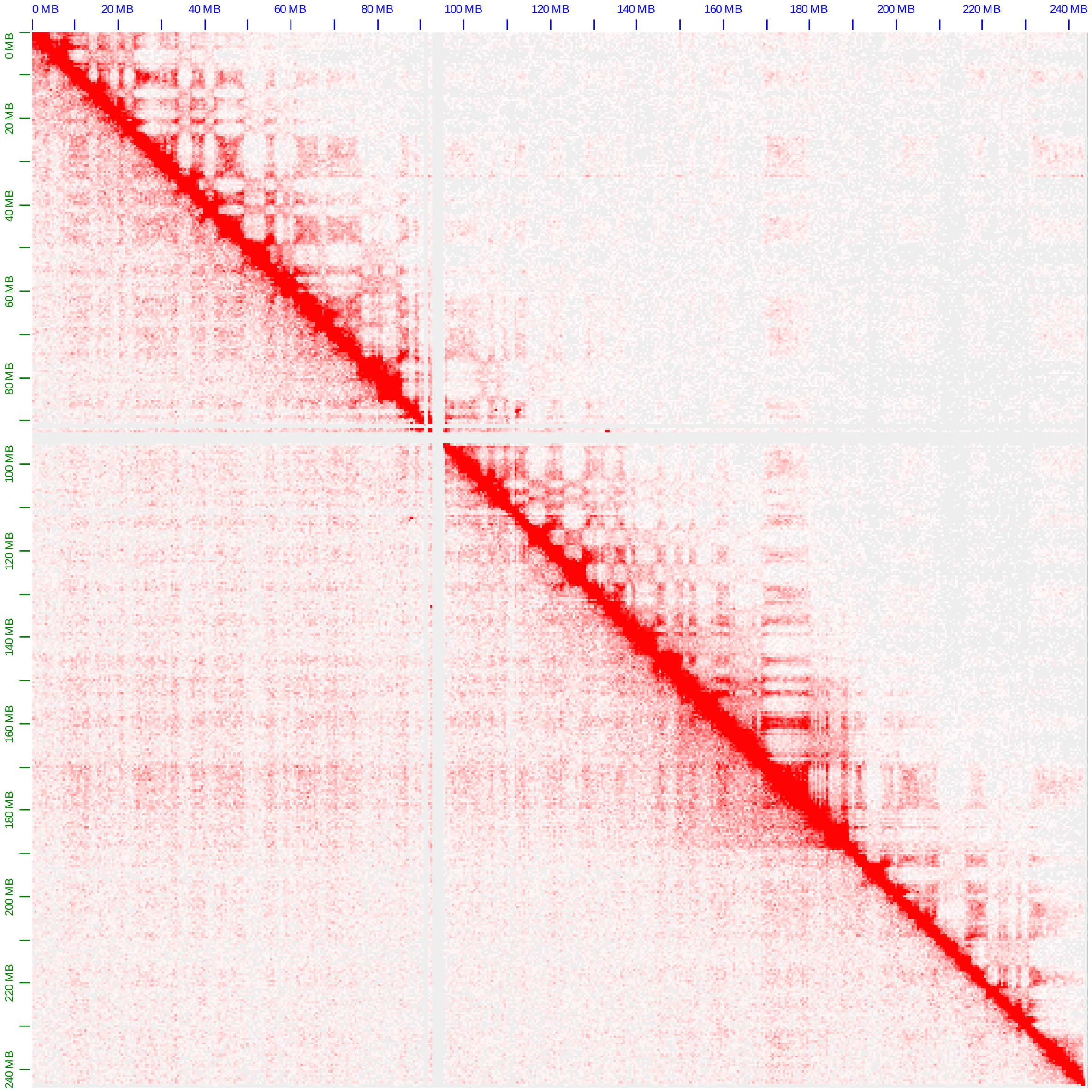

Above diagonal: Protocol Ramani et al., biotin fill-in
Below diagonal: Ma et al. (reanalyzed), K562, capture
Resolution: 500kb
Chromosome: chr2
Color scale: 30

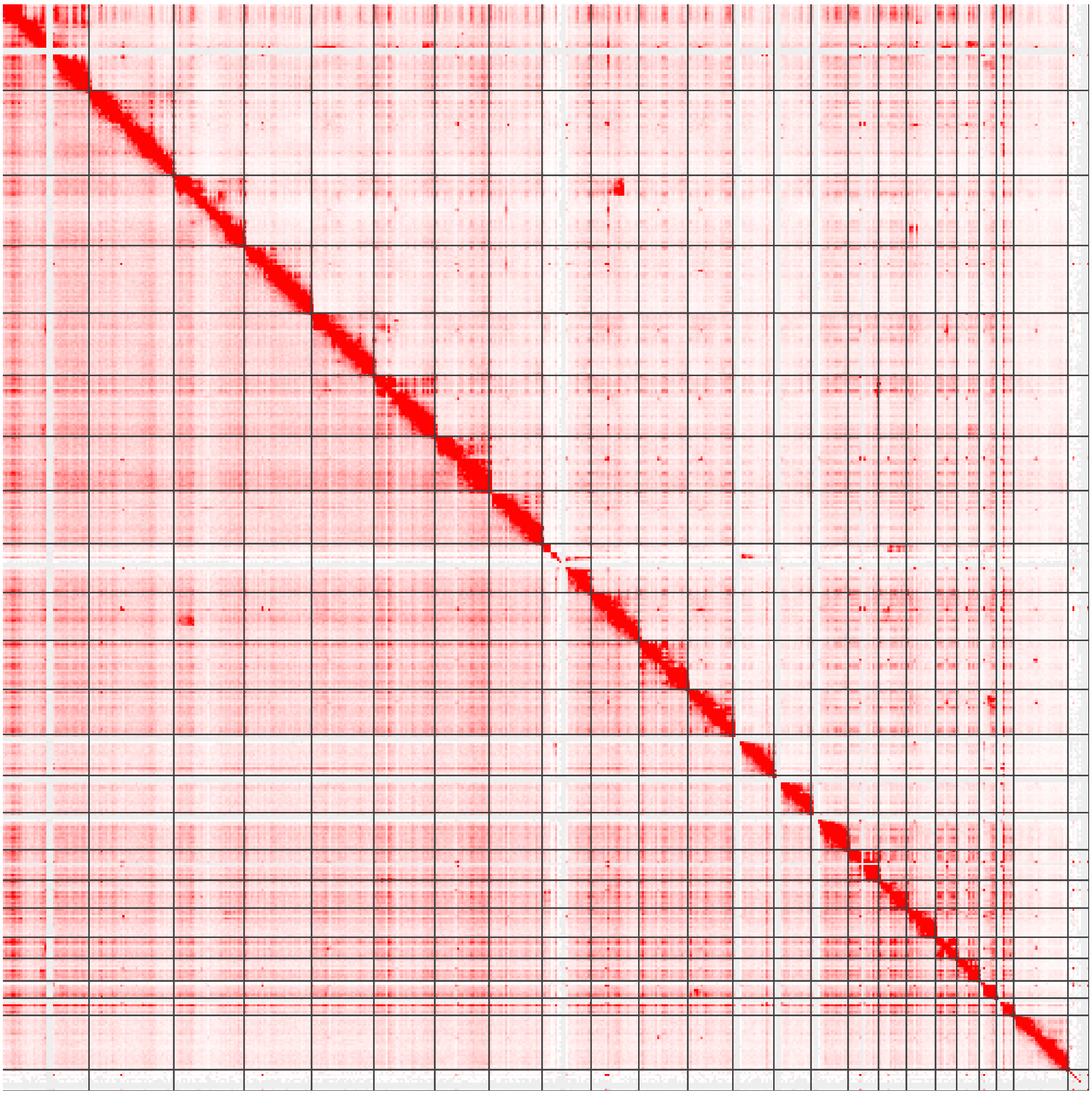

Above diagonal: Protocol Ramani et al., biotin fill-in
Below diagonal: Ma et al. (reanalyzed), K562, WGS
Full-genome view
Color scale: 366

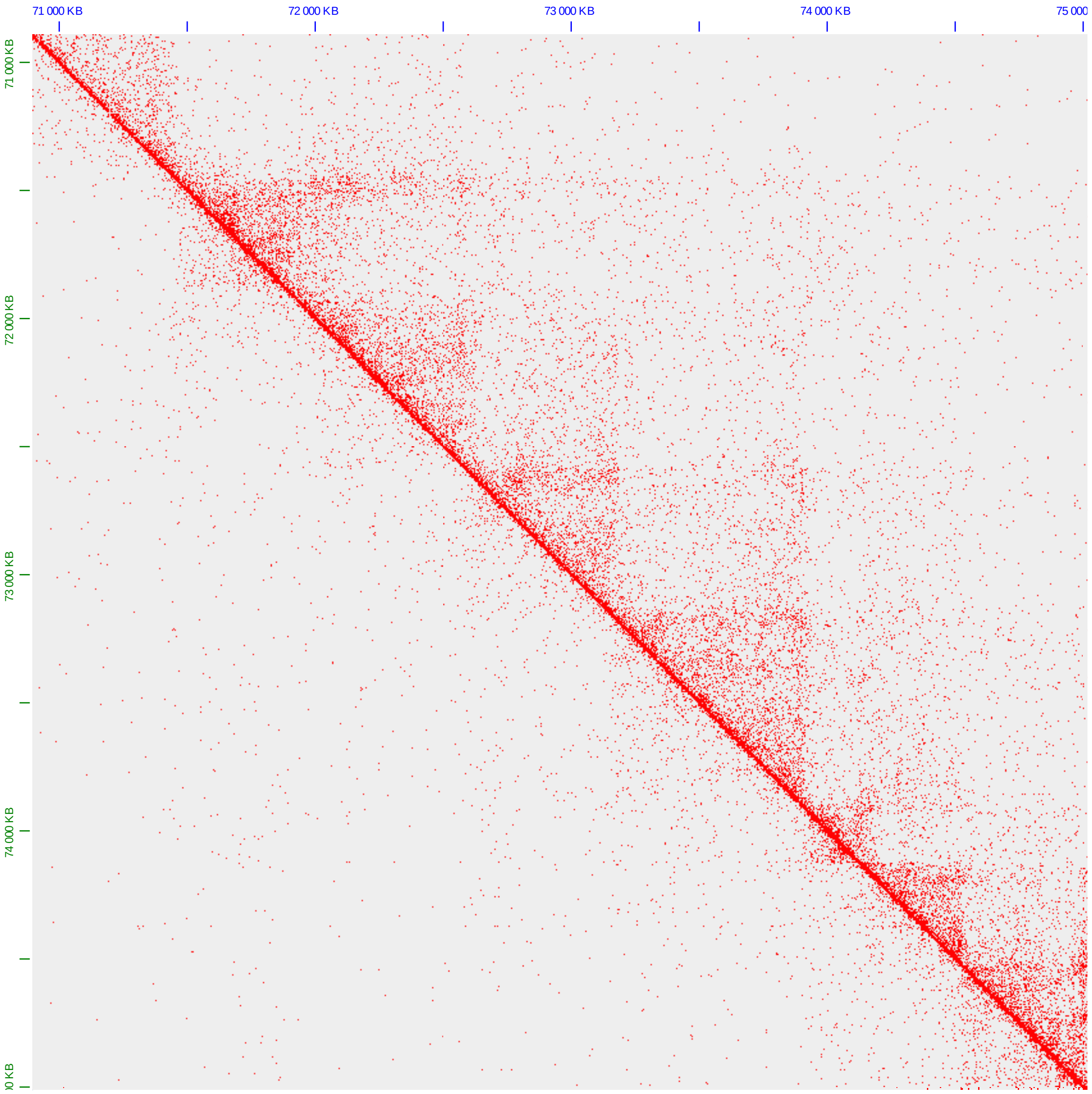

Above diagonal: Protocol Ramani et al., biotin fill-in
Below diagonal: Ma et al. (reanalyzed), K562, WGS
Resolution: 5kb
Chromosome: chr4
Color scale: 0

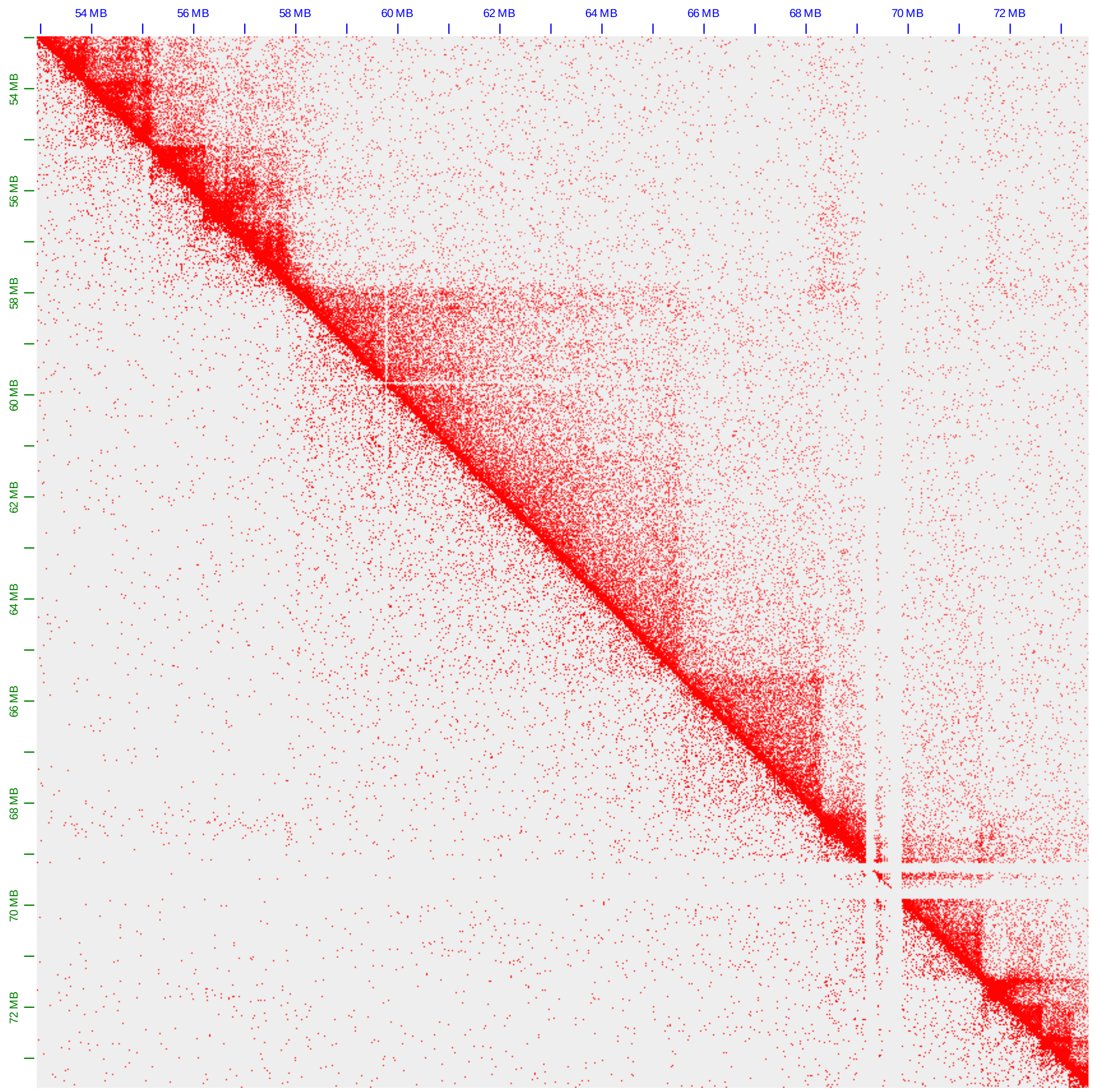

Above diagonal: Protocol Ramani et al., biotin fill-in
Below diagonal: Ma et al. (reanalyzed), K562, WGS
Resolution: 25kb
Chromosome: chr4
Color scale: 1

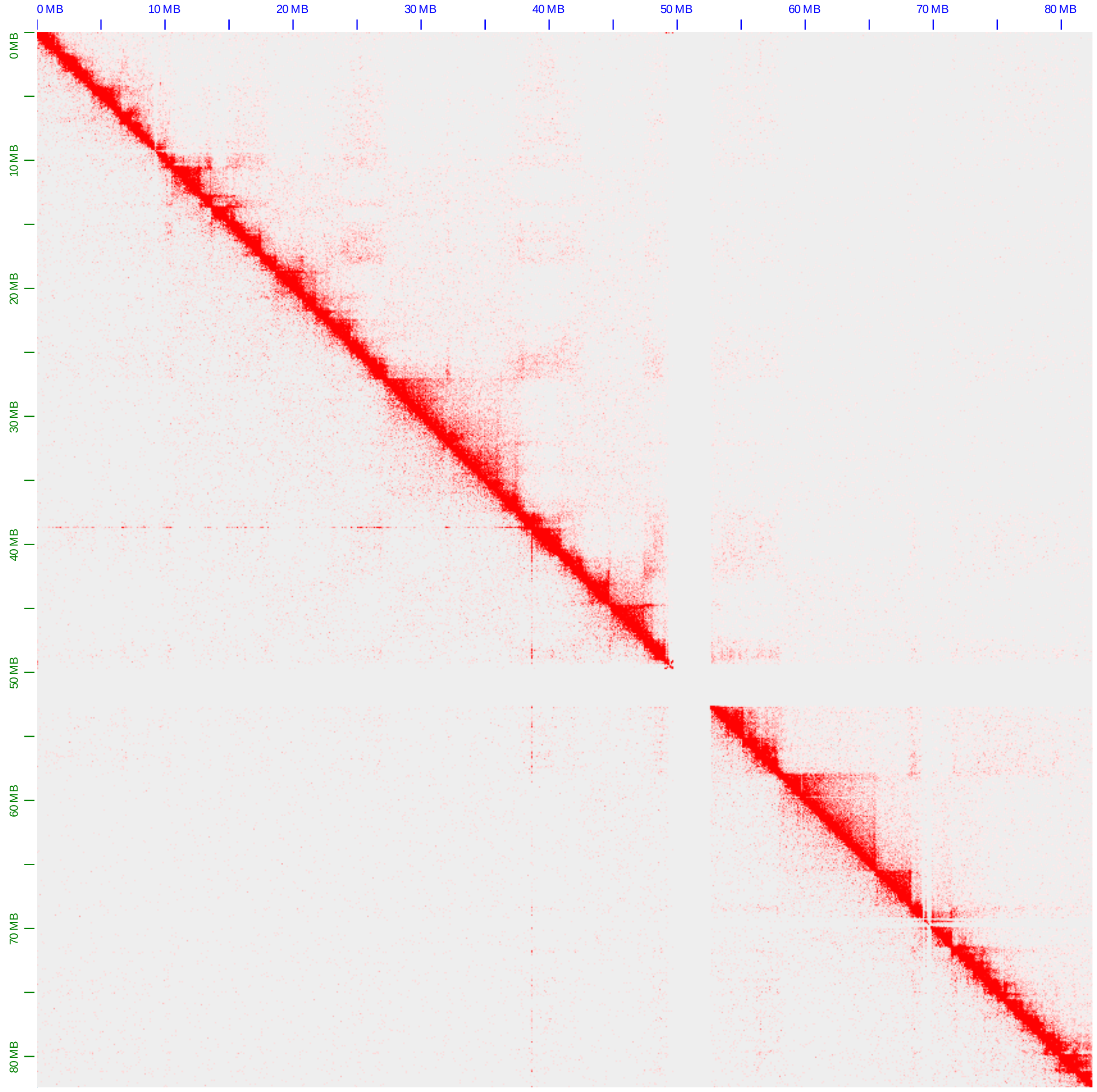

Above diagonal: Protocol Ramani et al., biotin fill-in
Below diagonal: Ma et al. (reanalyzed), K562, WGS
Resolution: 100kb
Chromosome: chr4
Color scale: 9

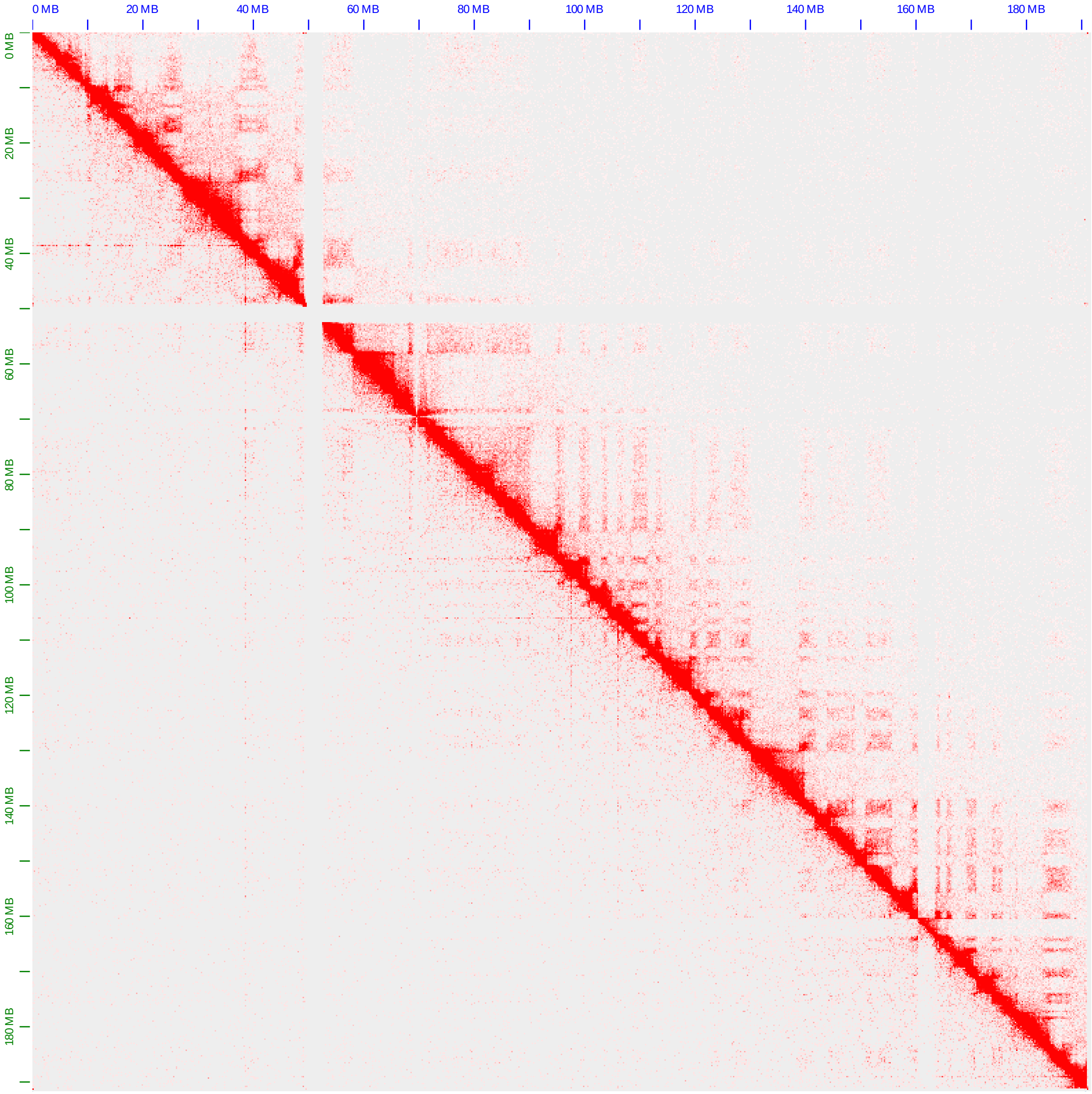

Above diagonal: Protocol Ramani et al., biotin fill-in
Below diagonal: Ma et al. (reanalyzed), K562, WGS
Resolution: 250kb
Chromosome: chr4
Color scale: 13

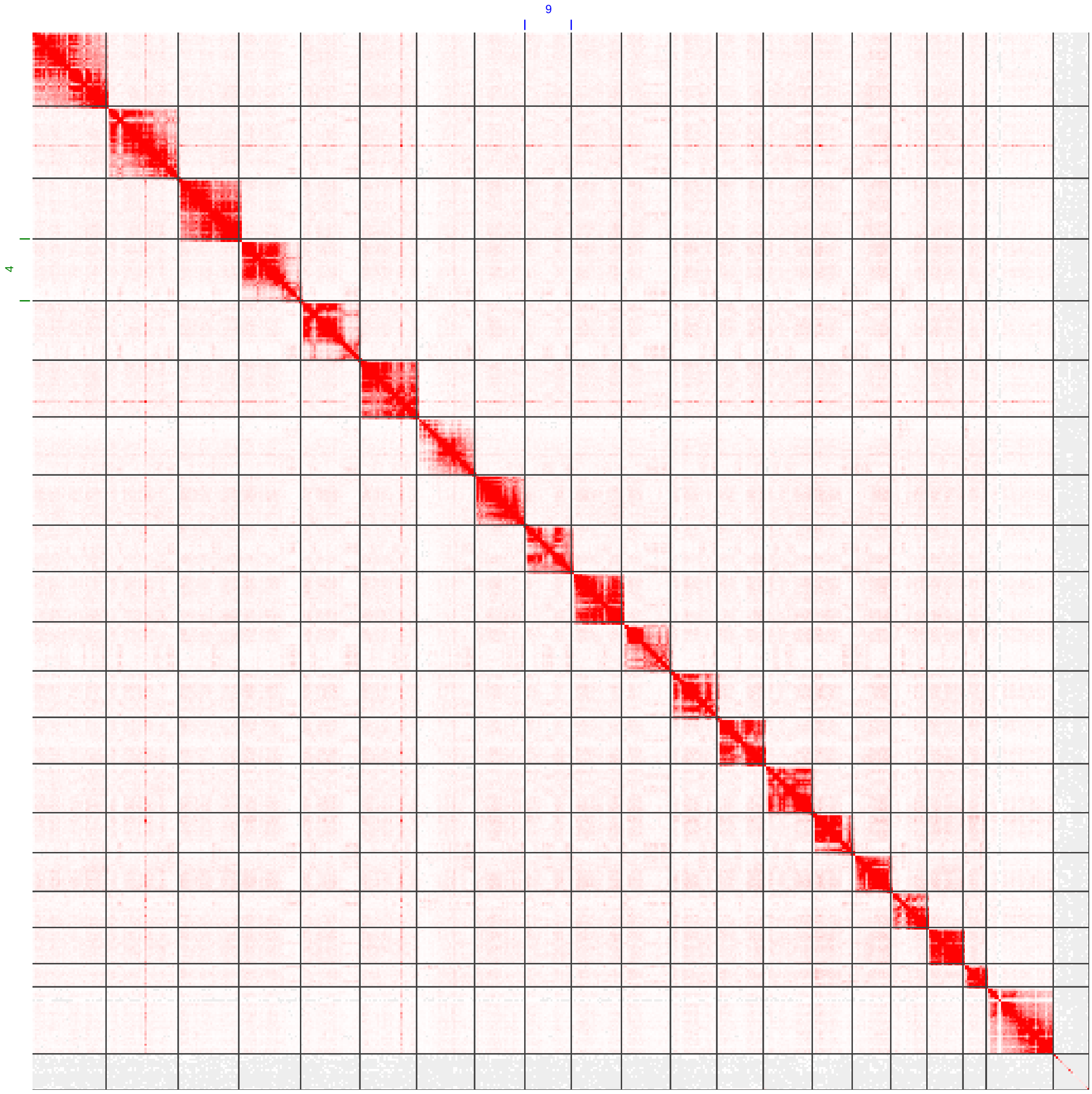

Sample: Ramani et al. (reanalyzed), M. musculus
Full-genome view
Color scale: 275

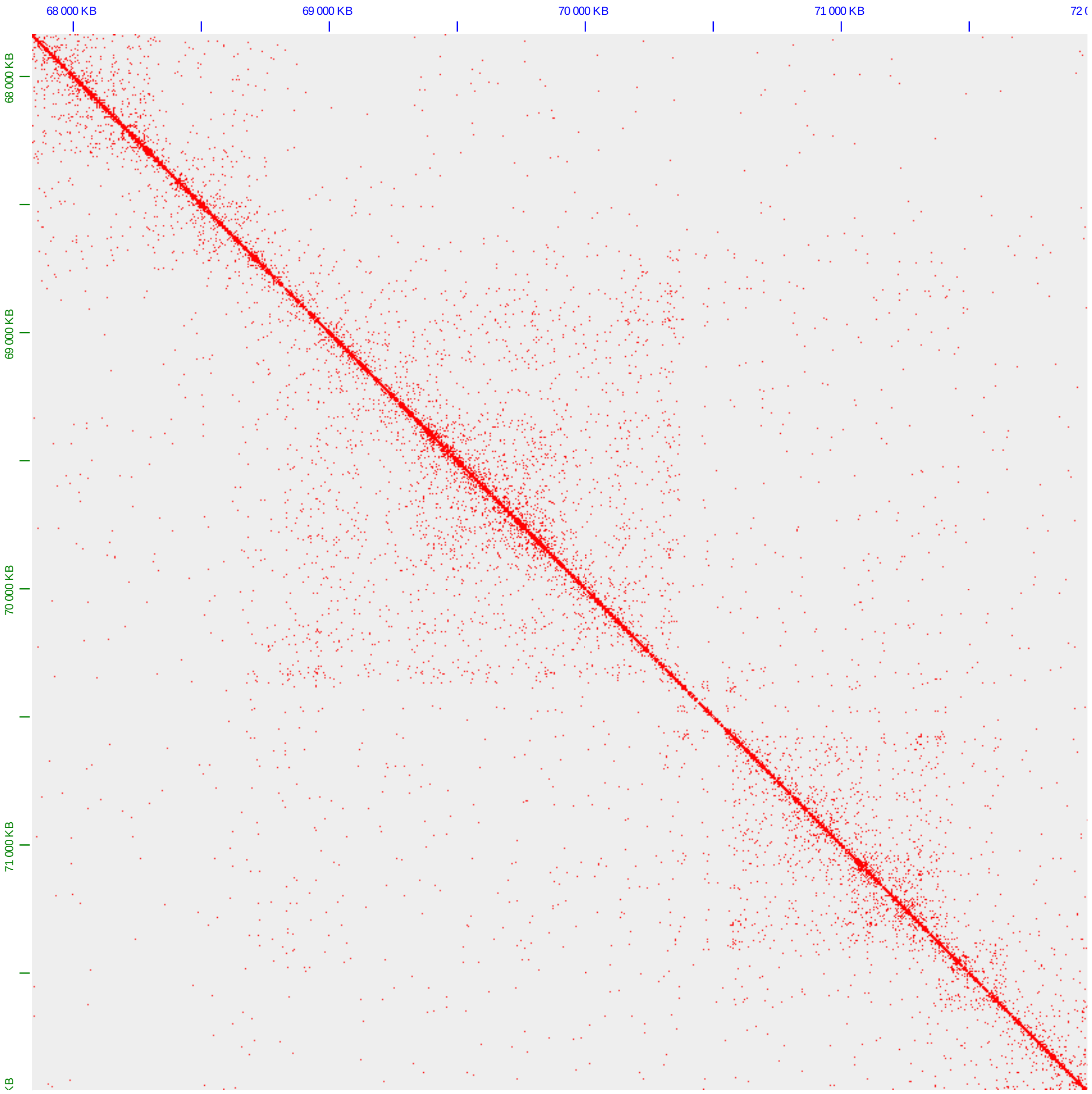

Sample: Ramani et al. (reanalyzed), M. musculus
Resolution: 5kb
Chromosome: chrX
Color scale: 0

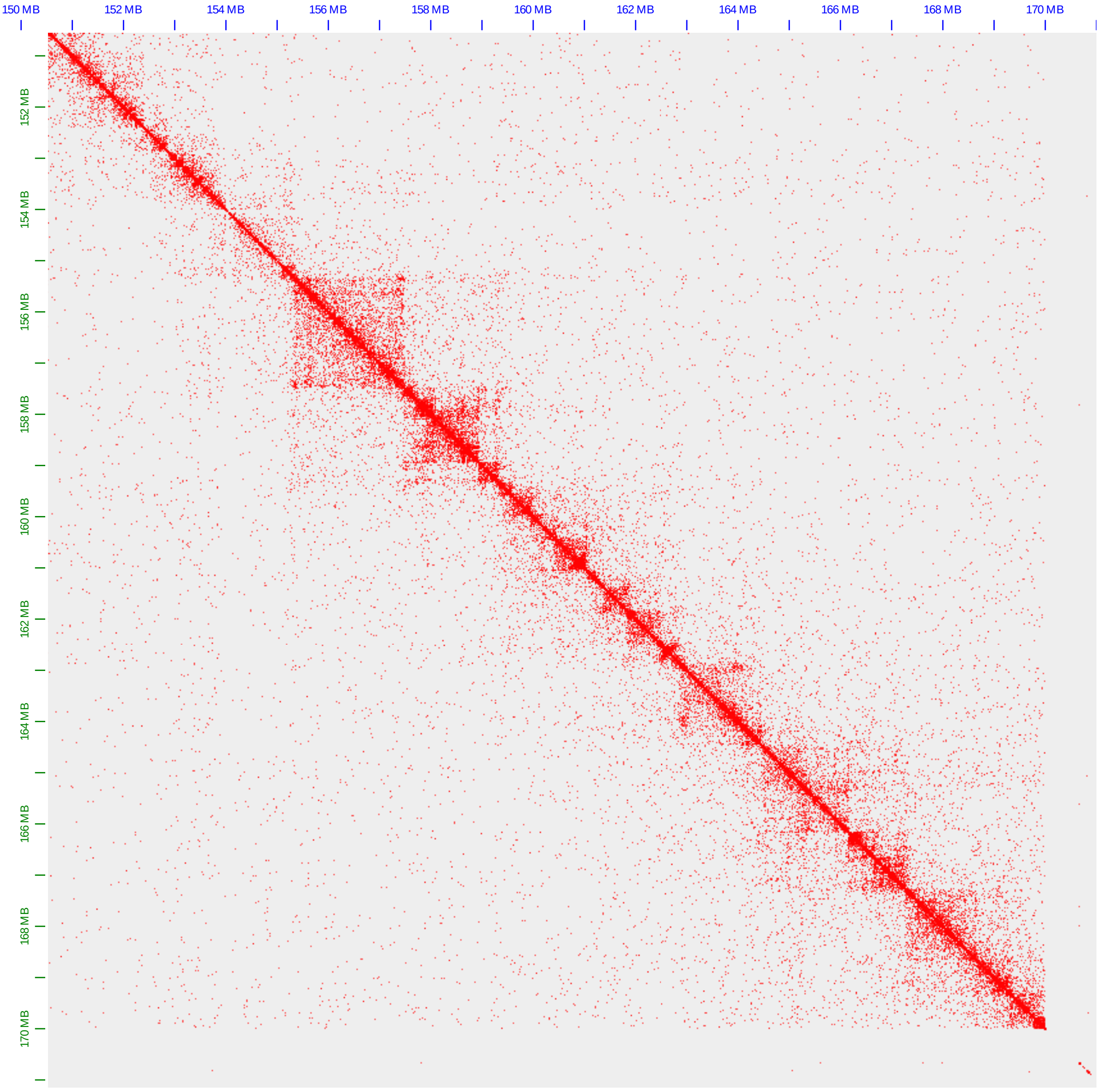

Sample: Ramani et al. (reanalyzed), M. musculus
Resolution: 25kb
Chromosome: chrX
Color scale: 1

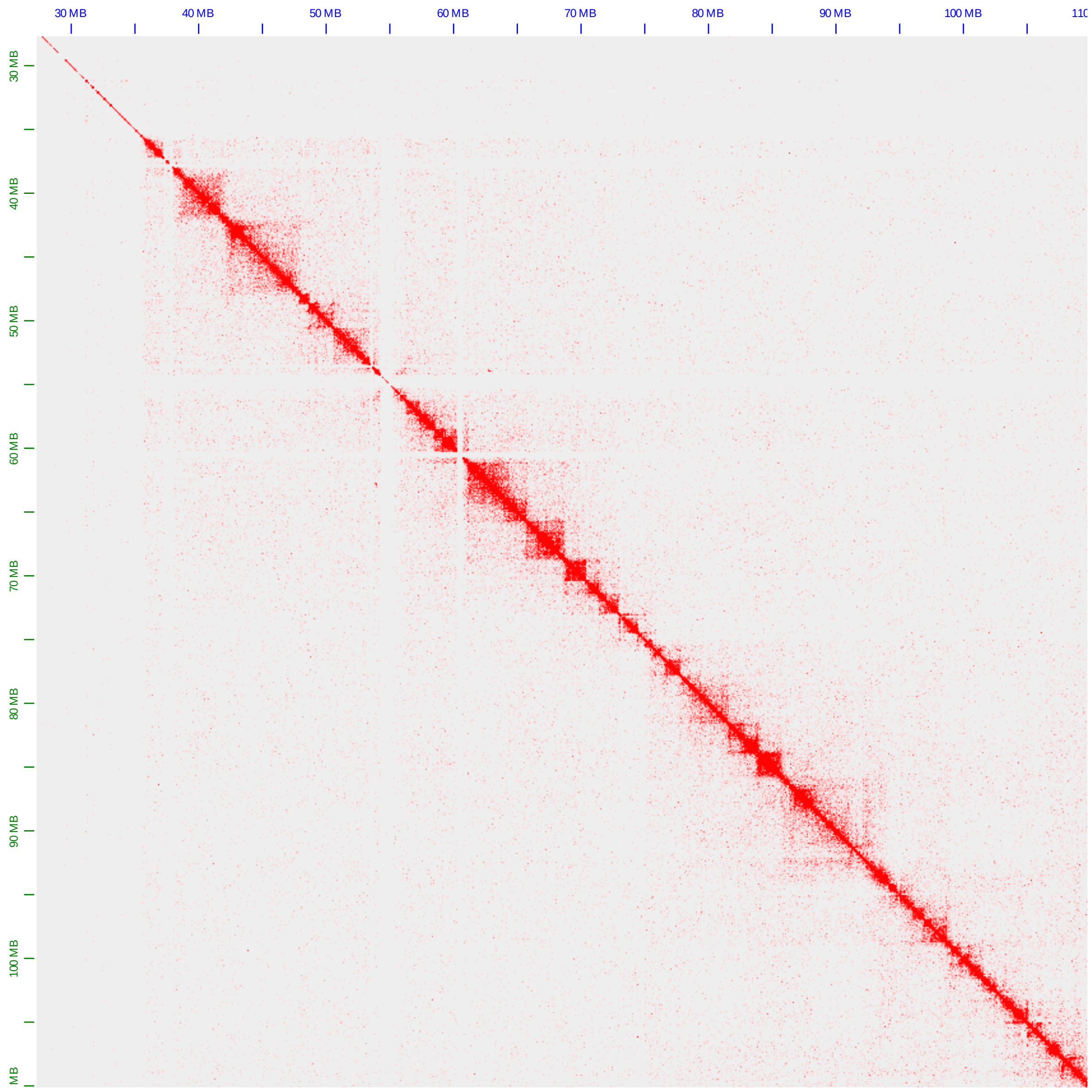

Sample: Ramani et al. (reanalyzed), M. musculus
Resolution: 100kb
Chromosome: chrX
Color scale: 5

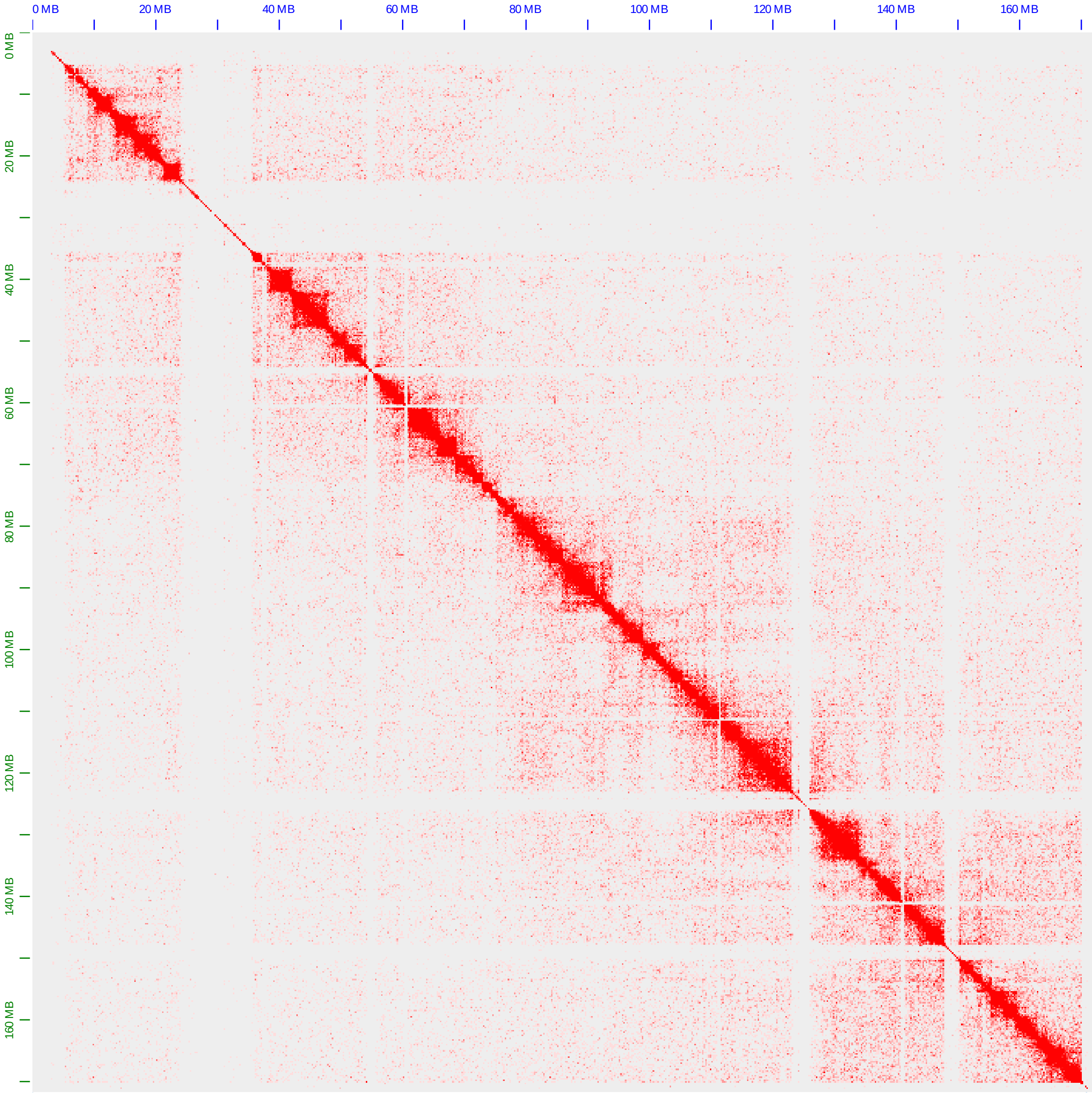

Sample: Ramani et al. (reanalyzed), M. musculus
Resolution: 250kb
Chromosome: chrX
Color scale: 7

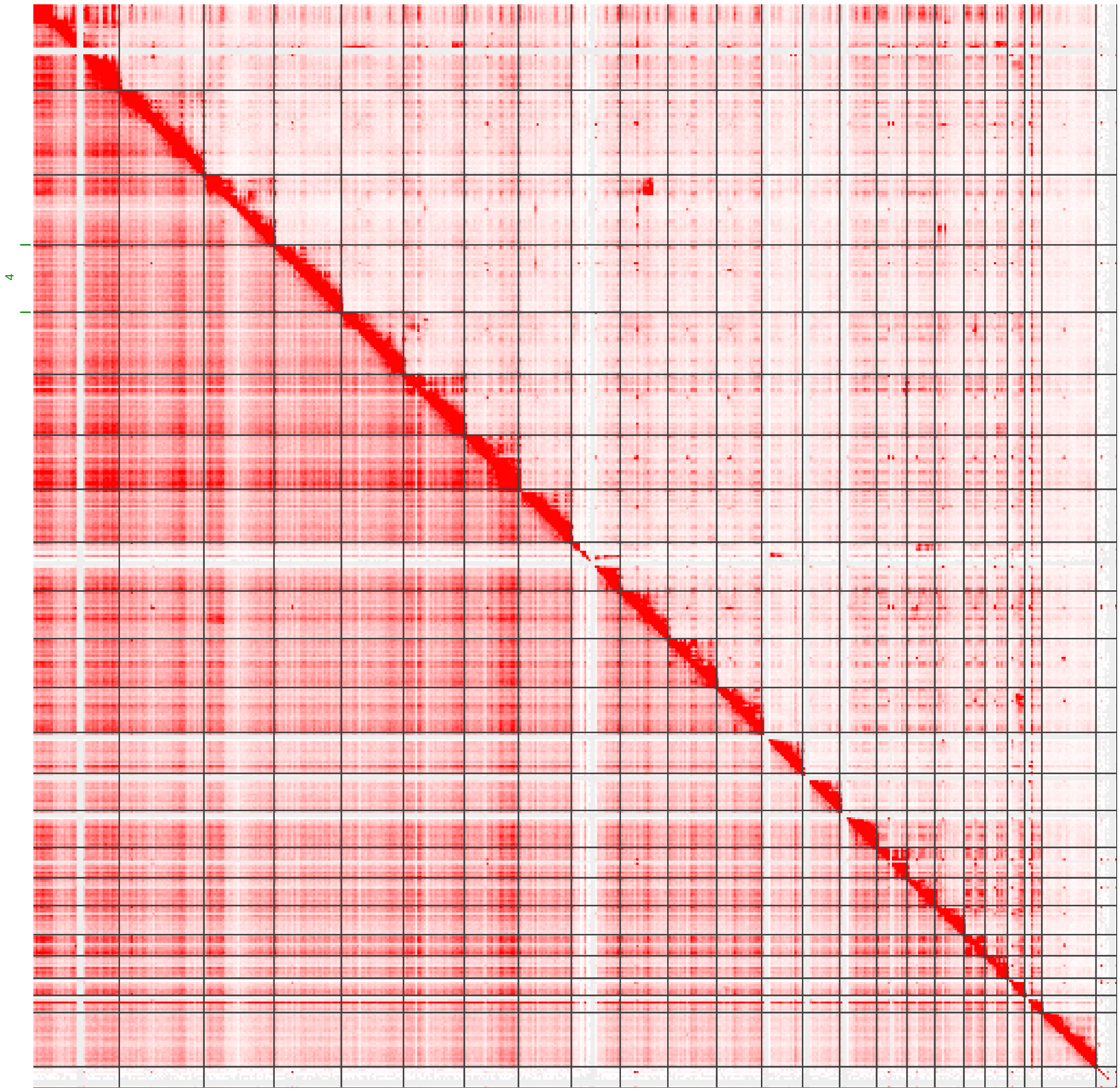

Above diagonal: Protocol Ramani et al., biotin fill-in
Below diagonal: Protocol: Ma et al., K562
Full-genome view
Color scale: 376

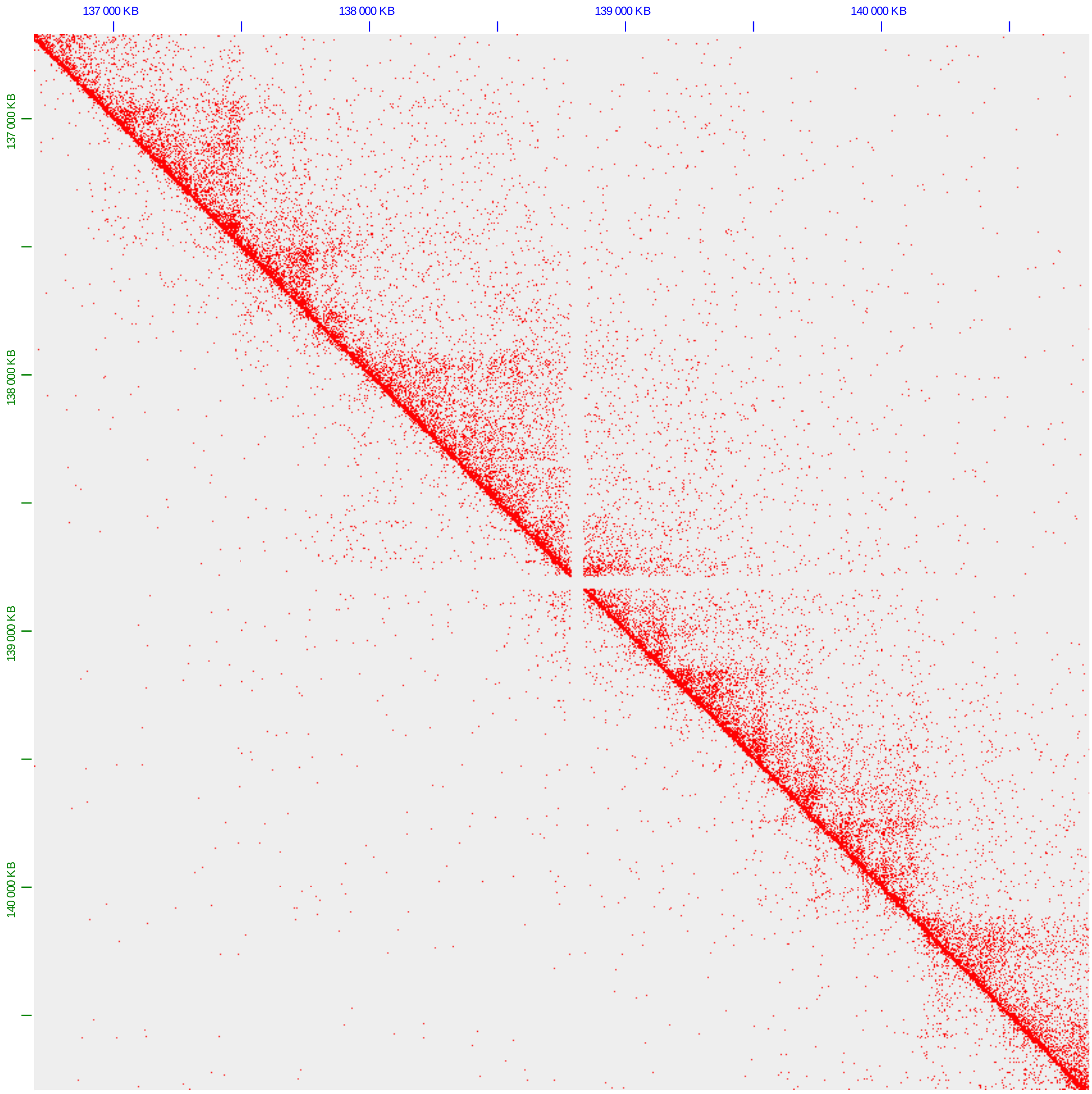

Above diagonal: Protocol Ramani et al., biotin fill-in
Below diagonal: Protocol: Ma et al., K562
Resolution: 5kb
Chromosome: chr5
Color scale: 0

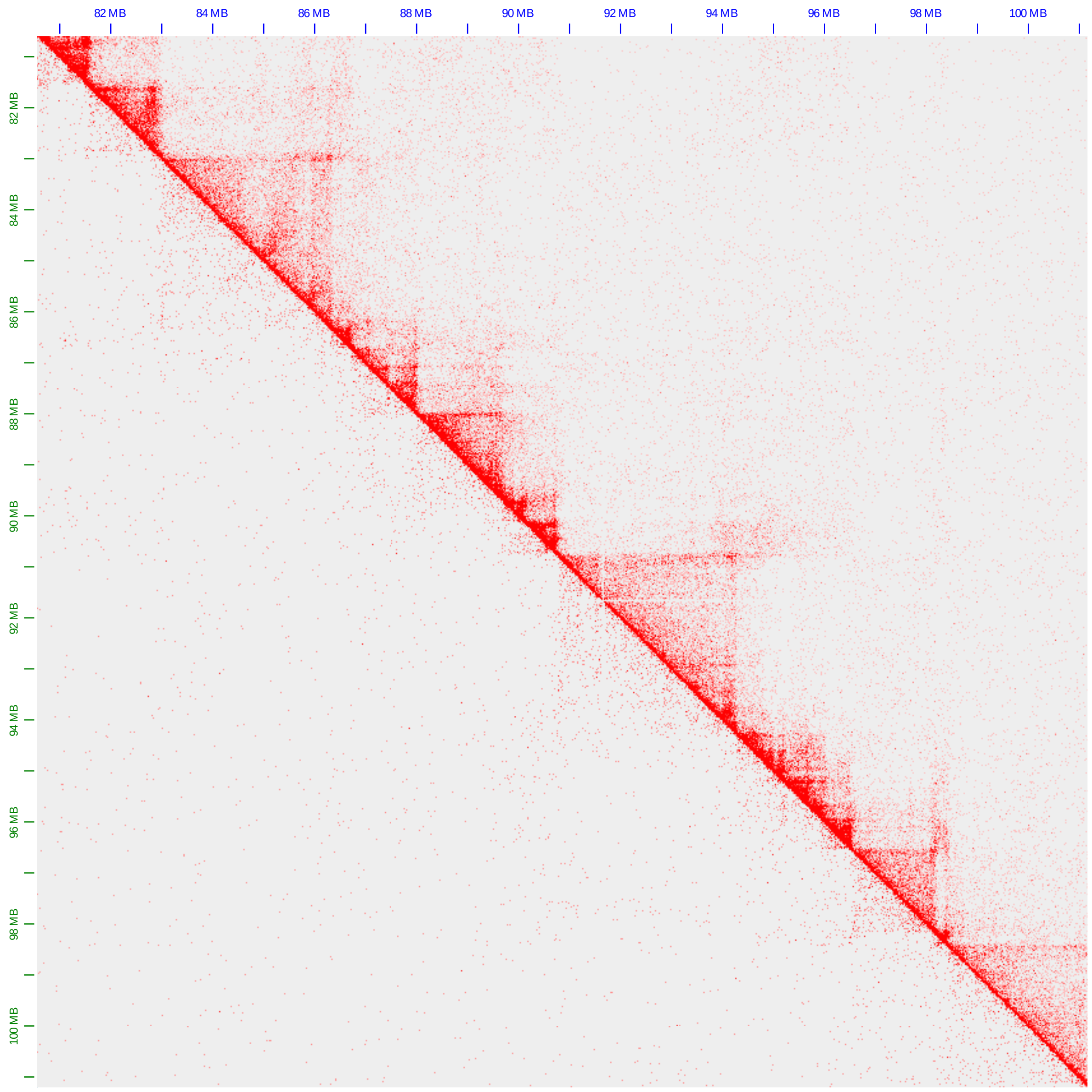

Above diagonal: Protocol Ramani et al., biotin fill-in
Below diagonal: Protocol: Ma et al., K562
Resolution: 25kb
Chromosome: chr5
Color scale: 3

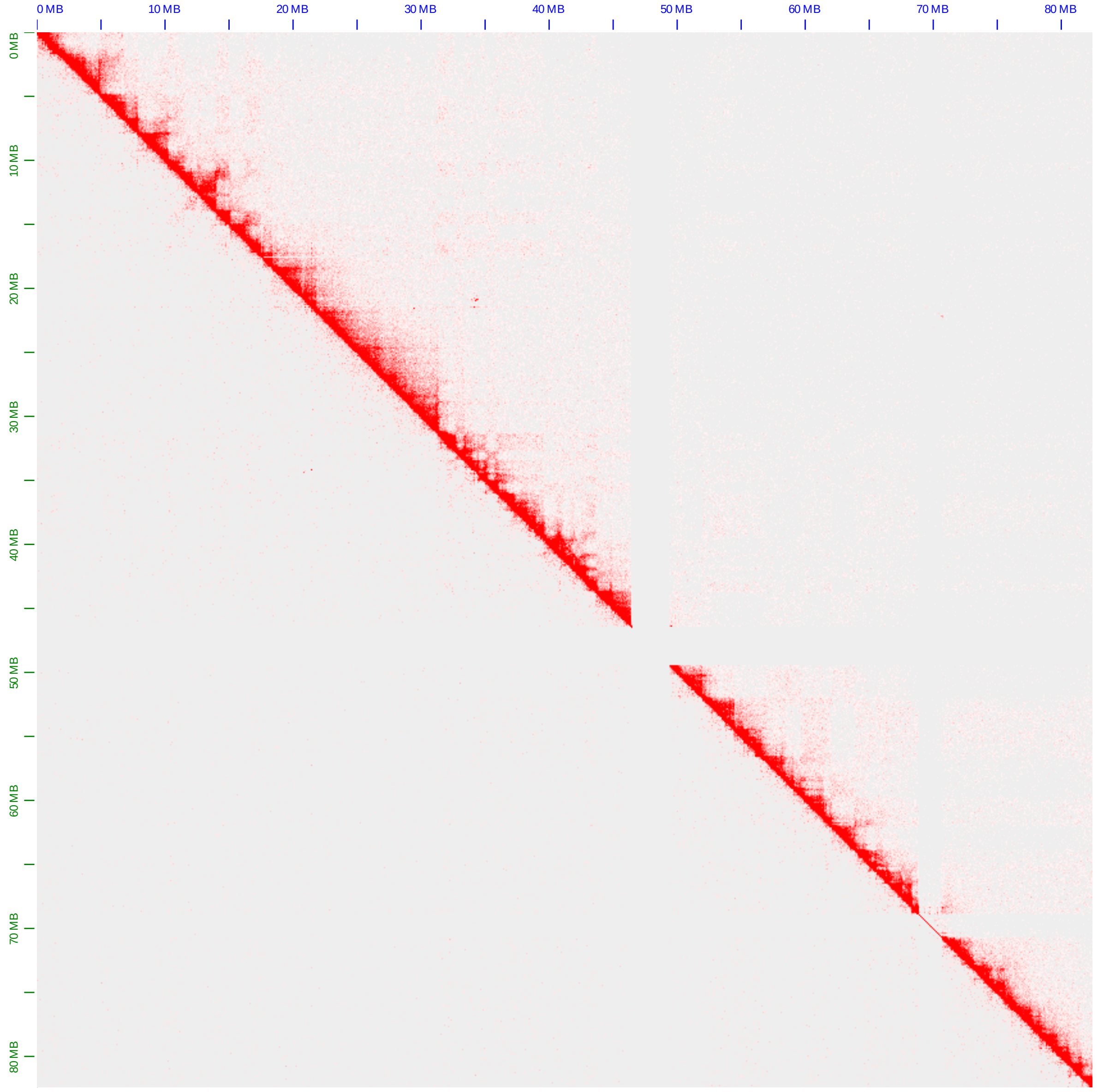

Above diagonal: Protocol Ramani et al., biotin fill-in
Below diagonal: Protocol: Ma et al., K562
Resolution: 100kb
Chromosome: chr5
Color scale: 14

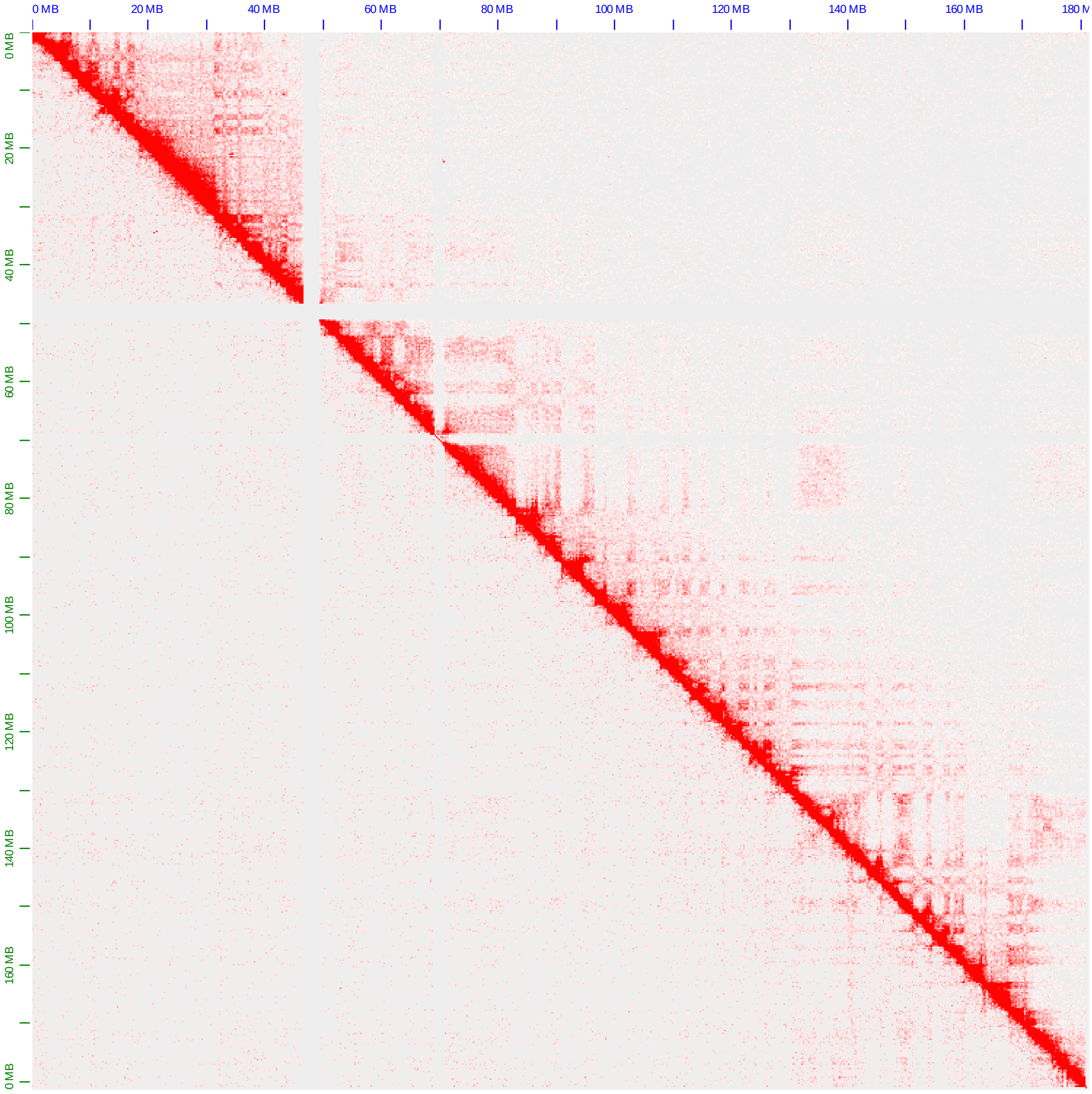

Above diagonal: Protocol Ramani et al., biotin fill-in
Below diagonal: Protocol: Ma et al., K562
Resolution: 250kb
Chromosome: chr5
Color scale: 14

Supplementary Figure 2. *FastContext* analysis of DNAse I Hi-C libraries

A

Protocol: Ma et al.

Rate [%]	Read structure	Hypothesis
16,52	-genome-	Unligated genome
7,86	-genome--bridge-gatc--egdirb--genome-	Unexpected ligation product
6,83	-genome--a-bridge-gatc--egdirb--genome-	Unexpected ligation product
6,41	-genome--bridge--gatc-egdirb-t--genome-	Unexpected ligation product
3,39	-genome--a-bridge-gatc--egdirb-t--genome-	Expected ligation product
2,28	-genome--bridge-gatc--egdirb--bridge-	Unexpected ligation product
2,26	-egdirb--bridge-gatc--egdirb--genome-	Unexpected ligation product
2,24	-genome--gatc-egdirb--genome-	Unexpected ligation product
2,12	-genome--gatc-egdirb-	Unexpected ligation product
1,97	-bridge-gatc--genome-	Unexpected ligation product
1,91	-genome--gatc-egdirb--bridge-	Unexpected ligation product
1,90	-genome--bridge-gatc--genome-	Unexpected ligation product
1,88	-genome--a-bridge-gatc--egdirb--bridge-	Unexpected ligation product, adapter multimers
1,83	-egdirb--bridge--gatc-egdirb-t--genome-	Unexpected ligation product, adapter multimers
1,47	-genome--egdirb--genome-	Unexpected ligation product
1,41	-genome--gatc-egdirb-t--genome-	Unexpected ligation product
1,40	-egdirb--bridge-gatc--genome-	Unexpected ligation product
1,29	-genome--bridge--genome-	Unexpected ligation product
1,27	-genome--a-bridge-gatc--genome-	Unexpected ligation product
0,97	-genome--bridge-gatc--egdirb-	Unexpected ligation product

B

Protocol: Ramani et al.

Rate [%]	Read structure	Hypothesis
19,03	-genome-	Unligated genome
6,98	-genome--a-bridge-gatc--egdirb-t--genome-	Expected ligation product
5,92	-genome--bridge--gatc-egdirb-t--genome-	Unexpected ligation product
5,83	-genome--a-bridge-gatc--egdirb--genome-	Unexpected ligation product
5,62	-genome--bridge-gatc--egdirb--genome-	Unexpected ligation product
4,59	-genome--a-bridge-gatc--genome-	Unexpected ligation product
4,05	-genome--bridge-gatc--genome-	Unexpected ligation product
3,14	-genome--gatc-egdirb-t--genome-	Unexpected ligation product
2,30	-genome--gatc-egdirb--genome-	Unexpected ligation product
0,99	-genome--bridge--genome-	Unexpected ligation product
0,99	-genome--egdirb--genome-	Unexpected ligation product
0,93	-genome--egdirb-t--genome-	Unexpected ligation product
0,72	-genome--a-bridge-gatc--egdirb-	Unexpected ligation product
0,71	-genome--a-bridge--genome-	Unexpected ligation product
0,64	-bridge--gatc-egdirb-t--genome-	Unexpected ligation product
0,59	--[6]--gatc-egdirb-t--genome-	Unexpected ligation product
0,54	-bridge-gatc--egdirb--genome-	Unexpected ligation product
0,51	-bridge-gatc--genome-	Unexpected ligation product
0,49	-genome--a-bridge-gatc--[6]--	Unexpected ligation product
0,47	-genome--bridge-gatc--egdirb-	Unexpected ligation product

Expected read structure

-genome-	-a-	-bridge-	-gatc-	-egdirb-	-t-	-genome-
NNNNNN	A	GCTGAGG	GATC	CCTCAGC	T	NNNNNN

FastContext analysis of DNAse I Hi-C libraries produced using protocols Ma et al. (A) and Romani et al. (B). Rate shows the percentage of reads with a specific read structure. The read structure may contain following elements (see figure legend in the bottom): bridge or blunt-

adapters sequences (-bridge- or -blunt-), reverse-complement of adapter sequences (-egdirb- or –tnulb-, respectively), bridge adapter overhang (-gatc-), and “a” or “t” nucleotides flanking the adapter sequences (-a- or -t-). Note that bridge/blunt adapters are ligated during adapter ligation step, and A/T-nucleotides are appended because of the A-tailing step (see protocols for details). Any other sequences longer than 6-bp are considered as genomic fragments (-genome-). Sequences shorted than 6-bp are labeled as k-mers (-[k]-).

Supplementary Table 1. Quality metrics of all DNAse I Hi-C datasets produced in this study

Sample name	Sample type	Library strategy	Reported pairs (% of total)*	Valid pairs (% of total)*	FR excess (% of valid)	Cis (% of valid)	Corrected DEnds estimation (% of valid)	Resolution, kb**
H1_rep1	human H1 cell line (PE-capture)	Ma et. al. (reanalyzed)	87	87	36	47	28	-
H1_rep2	human H1 cell line (PE-capture)	Ma et. al. (reanalyzed)	84	83	41	35	31	-
K562_rep1	human K562 cell line (PE-capture)	Ma et. al. (reanalyzed)	89	88	67	58	57	-
K562_rep2	human K562 cell line (PE-capture)	Ma et. al. (reanalyzed)	71	70	70	50	61	-
1	human blood	Protocol: Ma et. al.	79	60	74	29	39	-
2	human blood	Protocol: Ma et. al.	80	64	65	27	32	-
3	human blood	Protocol: Ma et. al.	87	70	58	48	44	-
4	human blood	Protocol: Ma et. al.	86	70	56	40	36	-
5***	human K562 cell line	Protocol: Ma et. al.	85	67	68	28	43	52
6	human blood	Protocol: Ma et. al.	79	66	66	23	28	-
7	human blood	Protocol: Ma et. al.	79	63	71	28	38	-
8	human blood	Protocol: Ma et. al.	81	64	65	27	33	-
9	human blood	Protocol: Ma et. al. + new blunt	92	66	74	59	34	-
10	human blood	Protocol: Ma et. al. + new blunt	89	72	57	34	21	-
11	human blood	Protocol: Ma et. al. + new blunt	89	71	62	33	25	-

12	human K562 cell line	Protocol: Ma et. al. + new blunt	89	66	58	25	24	-
13	human blood	Protocol: Ma et. al. + long linker	84	78	80	69	42	-
14	human blood	Protocol: Ma et. al. + long linker	86	81	64	81	16	-
15	human A549 cell line	Protocol: Ramani et. al. + biotin-fillin	93	87	68	80	-	-
16	human A549 cell line	Protocol: Ramani et. al. + biotin-fillin	92	86	60	78	-	-
17	human LNCap cell line	Protocol: Ramani et. al. + biotin-fillin	93	87	61	77	-	-
18	human LNCap cell line	Protocol: Ramani et. al. + biotin-fillin	92	86	66	75	-	-
19	human A549 cell line	Protocol: Ramani et. al. + long linker	88	80	41	75	16	-
20	human A549 cell line	Protocol: Ramani et. al. + long linker	89	81	48	71	27	-
21	human LNCap cell line	Protocol: Ramani et. al. + long linker	89	82	61	62	43	-
22	human blood	Protocol: Ramani et. al. + biotin-fillin	93	78	62	70	-	-
23	human blood	Protocol: Ramani et. al. + biotin-fillin	93	77	59	70	-	-
24	human blood	Protocol: Ramani et. al. + biotin-fillin	92	77	60	69	-	-
25	human blood	Protocol: Ramani et. al. + biotin-fillin	93	79	58	66	-	-
26	human blood	Protocol: Ramani et. al. + biotin-fillin	92	76	58	63	-	-
27	human blood	Protocol: Ramani et. al. + biotin-fillin	92	78	59	67	-	-
28	human blood	Protocol: Ramani et. al. + biotin-fillin	92	78	55	65	-	-
29	human blood	Protocol: Ramani et. al. + biotin-fillin	92	76	57	62	-	-
30	human K562 cell line	Protocol: Ramani et. al. + biotin-fillin	91	76	50	80	-	43
31	human LNCap cell line	Protocol: Ramani et. al. + biotin-fillin	93	79	57	76	-	-

32	human blood	Protocol: Ramani et al.	85	75	40	67	15	-
Ma et. al; k562***	human K562 cell line (PE-capture)	Ma et. al. (reanalyzed)	84	77	37	30	15	36
Ma et. al; k562***	human K562 cell line	Ma et. al. (reanalyzed)	90	55	66	58	13	56
Ramani et. al***	Mus musculus x Mus spretus brain cells	Ramani et. al. (reanalyzed)	81	30	39	88	2	79

*Note that percentage of the reported pairs indicates mapping efficiency, and percentage of valid pairs indicates the amount of PCR-duplicates, which in this study mainly depends on the sequencing depth. Both are shown as percentages of the total number of sequenced reads.

**Resolution was computed according to the approach described in Rao et al., 2014: "the smallest locus size such that 80% of loci have at least 1,000 contacts". Other metrics are explained in the main manuscript text.

***These data were subsamples to 81 936 439 reads to allow sequence-depth independent comparison

Supplementary Table 2. Mapping efficiency achieved for different alignment strategies

Sample name	Protocol	Hi-C pro (Bowtie2-based), reported pairs, %*	Bowtie2 after cutadapt split, reported pairs, %*	BWA (no preprocessing), reported pairs, %*
1	Protocol: Ma et. al.	37	48	79
2	Protocol: Ma et. al.	37	48	80
3	Protocol: Ma et. al.	63	67	87
4	Protocol: Ma et. al.	55	60	86
5	Protocol: Ma et. al.	55	62	85
6	Protocol: Ma et. al.	34	45	79
7	Protocol: Ma et. al.	34	49	79
8	Protocol: Ma et. al.	40	51	81
15	Protocol: Ramani et. al. + biotin-fillin	51	-	93
16	Protocol: Ramani et. al. + biotin-fillin	42	-	92
17	Protocol: Ramani et. al. + biotin-fillin	48	-	93
18	Protocol: Ramani et. al. + biotin-fillin	49	-	92

* Note: reported pairs are shown as percentage of the total number of sequenced reads.

Supplementary Note I. Supplementary DNAse I Hi-C protocols

Following we described several DNAse I Hi-C protocols used in this study. We note that the protocol which shows the best performance is described in the main article text and referred to as Protocol I.

Protocol II. DNase Hi-C with short blunt and bridge adapters

Note: this protocol describes preparation of DNAse I Hi-C libraries using biotinylated adapters ("Protocol: Ma et. al.", Ramani et al. this paper", "Protocol: Ma et. al. + new blunt").

Step 0. Preparation of adapter DNA

We use following adapter sequences:

Name adapter	Sequences 5' -> 3'	Protocol
Biotinylated Bridge	/5Phos/ GCTGAGGGA/iBiodT/C	Ma et. al. (reanalyzed), Protocol: Ma et. al., Ramani et al. this paper
	CCTCAGCT	
Blunt	GCTGAGGGAC	Ma et. al. (reanalyzed), Protocol: Ma et. al., Ramani et al. this paper
	CCTCAGC	
New Blunt	CAGTGGCGAC	Protocol: Ma et. al. + new blunt
	GCCACTG	

Long Linker (BAT-Hi-C linker)	/5Phos/ CGCGATATC/iBiodT/TATCTGACT<br>/5Phos/ GTCAGATAAGATATCGCGT	Protocol: Ramani et. al. + long linker

Adapter annealing (*volume 200 µl*):

0.1. Prepare *100 µM* stock of each oligs.
0.2. Mix following on ice:

Reagents	Amount per tube (µl)	Final
10X NEBuffer 2	20	1X
100 µM first oligs	80	40 µM
100 µM second oligs	80	40 µM
H2O	20	

0.3. Run following program:

- 95 °C — 5 min ramp 0.1 °C/second
- 75 °C — 2 min ramp 0.1 °C/second
- 65 °C — 2 min ramp 0.1 °C/second
- 50 °C — 2 min ramp 0.1 °C/second
- 37 °C — 2 min ramp 0.1 °C/second
- 20 °C — 2 min ramp 0.1 °C/second
- 4 °C — ∞

Keep annealed adapters on ice.

Protocol IIA. Protocol based on Ramani et al. ("Ramani et al., this paper")

Steps 1 and 2 were the same as in the Protocol I (Ramani et al. + biotin fill-in).

Step 3. End repairing (volume 200 µl)

3.1. Mix following on ice:

Reagents	Amount per tube (µl)	Final
NEBuffer 3.1	20	1X
dATP, 10 mM	1.5	75 µM
dTTP, 10 mM	1.5	75 µM
dGTP, 10 mM	1.5	75 µM
dCTP, 10 mM	1.5	75 µM
Klenow (5 U/µl)	10	50 U
H2O	64	

3.2. Add to the tube from the previous step.

3.3. Incubate at 23 °C on a thermomixer for 4 hrs with intermittent gentle shaking.

3.4. Add an equal volume of 20 PEG buffer (20% (wt/v) PEG-8000, 2.5M NaCl) to stop the reaction. Nuclei were bounded by the AMPure Beads, which have stayed in the reaction from Step 2, in these conditions.

3.5. Wash nuclei-beads mixture twice with 80% ethanol.

3.6. Resuspend in 100 µl H2O. AMPure Beads stayed in the following reaction.

Step 4. A-Tailing (volume 200 µl)

4.1. Mix following on ice:

Reagents	Amount per tube (µl)	Final
NEBuffer 2	20	1X
dATP, 10 mM	10	500 µM
Klenow EXO- (5 U/µl)	15	75 U
H2O	55	

4.2. Add to the tube from step 3.6.

4.3. Incubate at 37 °C on a thermomixer for 2 hrs with intermittent gentle shaking.

4.4. Add an equal volume of 20 PEG buffer (20% (wt/v) PEG-8000, 2.5 M NaCl) to stop the reaction. Nuclei were bounded by the AMPure Beads, which have stayed in the reaction from step 2, in these conditions.

4.5. Wash nuclei-beads mixture twice with 80% ethanol

4.6. Resuspend in 29 µl H2O. AMPure Beads stayed in the following reaction.

Step 5. Adapters ligation (volume 100 µl)

5.1. Mix following on ice:

Reagents	Amount per tube (µl)	Final
10x T4 ligase buffer	10	1X
10% Triton X-100	5	0.5%
50% PEG-4000	10	5%

BSA 100 mM	1	1 mM
annealed biotinylated adaptor 40 µM	20	8 µM
annealed blunt adaptor 40 µM	20	8 µM
T4 DNA ligase	5	

5.2. Add to the tube from step 4.6.

5.3. Incubate at 16 °C on a thermomixer at night with continuous shaking.

5.4. Add 5 µl 10% SDS and 95 µl H2O

5.5. Add 165 µl of 20 PEG buffer (20% (wt/v) PEG-8000, 2.5 M NaCl)

5.6. Wash nuclei-bead mixture once with 80% ethanol

5.7. Resuspend in 200 µl H2O.

5.8. Add 165 µl of 20 PEG buffer (20% (wt/v) PEG-8000, 2.5 M NaCl)

5.9. Wash nuclei-bead mixture once with 80% ethanol

5.10. Resuspend in 50 µl H2O.

Step 6. Phosphorylation (volume 100 µl)

6.1. Mix following on ice:

Reagents	Amount per tube (µl)	Final
10x T4 ligase buffer	10	1X

PNK (10 U/µl)	10	100
H2O	30	

6.2. Add to the tube from step 5.9.

6.3. Incubate at 37 °C on a thermomixer for 1 hrs with intermittent gentle shaking.

Step 7. In situ ligation (volume 1000 µl)

7.1. Mix following on ice:

Reagents	Amount per tube (µl)	Final
10x T4 ligase buffer	100	1X
10% Triton X-100	100	1%
25% PEG-8000	200	5%
BSA 100 mM	10	1 mM
T4 DNA ligase	20	
H2O	470	

7.2. Add to the tube from step 6.3.

7.3. Incubate at 16 °C on a thermomixer for at least 8 hrs (night is also appropriate) with continuous shaking.

Cross-link reversal and all subsequent steps as in the protocol “Ramani et al. + biotin fillin”.

Protocol IIB. Protocol which employs single long biotinylated linker (“Ramani et al. + long linker”)

Steps 0-4 were the same as in Protocol IIA. We used a single Long Linker (adapter) derived from BAT-Hi-C technique for this protocol.

Step 5. Ligation adapters and proximity ligation were performed in one step (volume 1000 µl)

5.1. Mix following on ice:

Reagents	Amount per tube (µl)	Final
10x T4 ligase buffer	100	1X
10% Triton X-100	100	1%
25% PEG-8000	200	5%
BSA 100 mM	10	1 mM
annealed adaptor 100 µM	6	600 nM
T4 DNA ligase	20	
H2O	535	

5.2. Add to the tube from the previous step.

5.3. Incubate at 16 °C on a thermomixer for at least 8 hrs (night is also appropriate) with continuous shaking.

Cross-link reversal and all subsequent steps are as in the protocol “Ramani et al. + biotin fillin”.

Protocol IIC. Protocol based on Ma et al. (“Protocol: Ma et. al.”)

- Step 1 was the same as in Protocol I
- Step 2 was the same as in the Protocol I till the Step 2.3. Then:

1. The pellet of nucleus was resuspended in 1 ml TE lysis buffer (50 mM Tris.HCl (pH7.0), 1 mM EDTA, 1% SDS), incubated at 37 °C for 10 min and centrifuged at 2500 g for 5 minutes.
2. The pellet resuspended in 400 µl of DNase I digestion buffer (5 mM Tris-HCl (pH 7.5 at 25 °C), 0.05 mM CaCl, 2. 5 mM MnCl2) and incubated at 37 °C for 10 min.
3. The chromatin was digested by 1-8 U DNase I (Thermo Scientific) at RT strictly during 5 min. The reaction was stopped immediately after 5 min by 40 µl Stop buffer (125 mM EDTA, 2.5% SDS).
4. Nuclei were bounded by 2x volume of AMPure Beads (Beckman Coulter) washed twice with 80% ethanol and resuspended in 100 µl H2O. AMPure Beads stayed in the following reaction.

All subsequent steps were as in the Protocol IIA (“Ramani et al. this paper”).

Protocol IID. Modification of the protocol bt Ma et al. which prevents adapter oligonucleotides multimerization (Protocol: Ma et. al. + new blunt)

The differences from the protocol II.C (“Protocol: Ma et. al.”) are:

- Incubation in TE lysis buffer at 37 °C during 30 min
- Using oligs New Blunt together with Biotinylated Bridge
